# A classic approach for determining genomic prediction accuracy under terminal drought stress and well-watered conditions in wheat landraces and cultivars

**DOI:** 10.1371/journal.pone.0247824

**Published:** 2021-03-05

**Authors:** Morteza Shabannejad, Mohammad-Reza Bihamta, Eslam Majidi-Hervan, Hadi Alipour, Asa Ebrahimi

**Affiliations:** 1 Department of Plant Breeding and Biotechnology, Faculty of Agricultural Sciences and Food Industries, Science and Research Branch, Islamic Azad University, Tehran, Iran; 2 Department of Agronomy and Plant Breeding, Faculty of Agricultural Sciences and Engineering, College of Agriculture and Natural Resources, University of Tehran, Tehran, Alborz, Iran; 3 Department of Plant Production and Genetics, Faculty of Agriculture and Natural Resources, Urmia University, Urmia, Iran; North Dakota State University, UNITED STATES

## Abstract

The present study aimed to improve the accuracy of genomic prediction of 16 agronomic traits in a diverse bread wheat (*Triticum aestivum* L.) germplasm under terminal drought stress and well-watered conditions in semi-arid environments. An association panel including 87 bread wheat cultivars and 199 landraces from Iran bread wheat germplasm was planted under two irrigation systems in semi-arid climate zones. The whole association panel was genotyped with 9047 single nucleotide polymorphism markers using the genotyping-by-sequencing method. A number of 23 marker-trait associations were selected for traits under each condition, whereas 17 marker-trait associations were common between terminal drought stress and well-watered conditions. The identified marker-trait associations were mostly single nucleotide polymorphisms with minor allele effects. This study examined the effect of population structure, genomic selection method (ridge regression-best linear unbiased prediction, genomic best-linear unbiased predictions, and Bayesian ridge regression), training set size, and type of marker set on genomic prediction accuracy. The prediction accuracies were low (-0.32) to moderate (0.52). A marker set including 93 significant markers identified through genome-wide association studies with *P* values ≤ 0.001 increased the genomic prediction accuracy for all traits under both conditions. This study concluded that obtaining the highest genomic prediction accuracy depends on the extent of linkage disequilibrium, the genetic architecture of trait, genetic diversity of the population, and the genomic selection method. The results encouraged the integration of genome-wide association study and genomic selection to enhance genomic prediction accuracy in applied breeding programs.

## Introduction

The world wheat production in 2020 is estimated at 762.7 million tons [1]. Bread wheat (*T*. *aestivum*) has approximately 10.67 grams of proteins and 47.54 grams of carbohydrates (per 100 grams of seeds), which is remarkably higher than other cereals and makes bread wheat one of the most strategic crops [2]. Nonetheless, it should be noted that the quality of bread wheat amino acids is not good enough for the human body and should be consumed along with other sources of proteins [3].

Wheat is a highly adapted plant species which can grow between the latitudes of 30° to 60°N and 27° to 40°S [4]. In the Middle East, drought stress normally occurs at the end of the growing season when the spike has already appeared. Drought and heat stresses can dramatically decrease yield in this phase of growth [5]. Drought stress affects many vital biological processes in plants such as photosynthesis, respiration, and metabolism [6, 7]. In the Persian plateau, where most of the climate zone is arid or semi-arid, farmers are very well-trained during the centuries to deposit rainwater throughout spring for irrigating farms at the end of the growing season when there is no rain during the seed development stage [8]. The Persian farmers irrigate their farms two to four more times with the stored water after spike appearance to avoid yield loss due to late-season drought stress [8].

Genetic studies have identified many quantitative trait loci (QTL) for wheat traits (https://triticeaetoolbox.org; http://plants.ensembl.org). However, some early known QTL were not suitable for identifying candidate genes or even marker-assisted selection (MAS) due to the unsatisfactory marker density or limited recombination rate [9]. After introducing genotyping-by-sequencing (GBS) [10] and implementing wheat genome sequencing projects [11, 12], many single nucleotide polymorphisms (SNPs) of complex traits are found using the genome-wide association study (GWAS) [5]. GWAS can identify QTL with the use of high marker density in complex genomes of diverse or breeding populations [9]. However, these SNPs can be putative with minor allele effects [5]. All recent studies have shown that agronomic traits can be significantly affected by environmental stresses [13]. Therefore, studying the genetic basis of agronomic traits under stress conditions will help accelerating genetic gain in breeding programs.

Genomic prediction (GP) [14] will boost the speed and efficiency of breeding programs by increasing selection accuracy and reducing time cycles [15]. Genomic selection (GS) produces a genomic estimated breeding value (GEBV) using all minor and major effects QTL in the genome, so that candidate genes can be selected by genotyping before phenotyping [15]. GP uses all markers within a model to train a prediction model in the training set (TS) which includes all genetic effects, without considering how minor the genetic effects are [8, 15]. The model will be applied to a validation set (VS) to estimate the accuracy of GP. The genetic artichature of traits, population structure, GS method, TS and marker set (MS) are major factors that can alter GP accuracy [15–18]. Many studies have reported moderate or high GP accuracy for quantitative traits in different populations of wheat (*Triticum aestivum* L.) [15], rice (*Oryza sativa* L.) [17], oat (*Avena sativa* L.) [19], maize (*Zea mays* L.) [20], switchgrass (*Panicum virgatum* L.) [21], barley (*Hordeum vulgare* L.) [22], and wheatgrass (*Thinopyrumn intermedium*) [23].

The present study determines GP accuracy using alleles derived from a mixed population of 87 cultivars and 199 landraces of Iran bread wheat germplasm. The goal was to optimize GP accuracy using different population structures, GS methods, TS sizes and types of MSs for 16 agronomic traits under terminal drought stress (TDS) and well-watered (WW) conditions.

## Materials and methods

### Plant materials and field trials

A collection of two hundred and eighty-six Iran breed wheat accessions including 199 landraces (collected during 1931–1968 in Persian plateau) and 87 cultivars (released during 1942–2014), was kindly provided by the University of Tehran (UT) and Seed and Plant Improvement Institute (SPII), Karaj, Iran. The detailed information about the landraces and cultivars is given in S1 and S2 Tables in S1 File. The experiments were carried out at the Kheirabad Agricultural Research Station (36°31’51.7"N and 48°45’29.9"E) in the Zanjan province and the Agricultural Research Farm of Karaj Islamic Azad University (35°43’44.1"N and 50°49’44.6"E) in Alborz province during the 2017–2018 cropping season using two separated alpha lattice designs in each location with two replications for each experimental design. The plots were 1 m in length, 1 m in width, and 0.5 m apart. Drip irrigation method was used for watering with the use of 2 tapes for each plot. Irrigation was conducted every ten days till the end of the spike appearance of all genotypes. When, some genotypes were in the seed development stage, TDS was inducted by terminating irrigation for one design in each location whereas another design was WW three more times. This issue occurs in some parts of the Persian plateau and the Middle East, naturally. Weather conditions were recorded during the cropping season (S1 Fig in S2 File). Both Zanjan and Alborz provinces are located in a cold semi-arid climate zone.

### Genotyping and quality control

The genotyping-by-sequencing (GBS) [10] method was used for DNA fingerprinting. The DNA extraction and library construction have been previously described for this collection [24]. The Trait Analysis by aSSociation Evolution and Linkage (TASSEL) software [25] was used to use the Universal Network Enabled Analysis Kit (UNEAK) pipeline [26] for SNP calling. The W7984 genome was used as the reference genome. The call success rate was greater than 85%. SNPs with a missing rate of > 20% were ruled out. SNPs with a minor allele frequency (MAF) < 5% were excluded as well. Unanchored SNPs were removed too. The remaining missing data were imputed using the LD KNNi method in TASSEL software [25]. Finally, a total of 9047 SNPs were used for further analysis.

### Population structure and kinship

The population structure was evaluated by the Bayesian clustering approach with the use of an admixture model in STRUCTURE software [27]. The number of subpopulations (K) was assessed with the use of 10000 burn-in and 10000 Markov Chain Monte Carlo (MCMC) for K = 1 to 10 in 10 independent runs. The best K value was estimated by ΔK statistic [28] in the structure harvester website (http://taylor0.biology.ucla.edu/structureHarvester). Two subpopulations (SBP-I and SBP-II) were identified within the association panel. The SNP calling was performed for each subpopulation, and 7714 SNPs for SBP-I and 5873 SNPs for SBP-II were identified. The 4785 markers were common between SBP-I and SBP-II, which were systematically separated for further analysis. The population structure matrix (Q-matrix) was obtained for the association analysis of the whole population. In addition, principal component analysis (PCA) was conducted on the SNP data set with the *prcomp* function using the tidyverse [29] package in the R environment. The first three PCs were plotted versus each other using the plotly [30] package to have a comprehensive perspective of the population. Also, a pairwise kinship coefficient matrix (K-matrix) that estimates the probability of the recent co-ancestry between genotypes [31] was achieved by the EMMA algorithm [32] embedded in the Genomic Association and Prediction Integrated Tool (GAPIT) [33] package in the R environment using the complete SNP data set.

### Molecular markers and linkage disequilibrium (LD)

The distribution of molecular markers and LD estimates was calculated for the whole association panel (WAP) and each subpopulation, separately. LD among markers was estimated for each chromosome using the full matrix option in TASSEL software [25]. Pairwise LD was measured as a squared correlation coefficient of alleles (*r*^2^) [34]. The cut-off line (*r*^2^ > 0.02) was chosen following Sukumaran et al. [35]. The percentage of marker pairs and LD estimates above the critical LD was determined for each chromosome and genome. Meanwhile, pairwise LD estimates were plotted versus the genetic distance (cM), and then the LD decay curve line was fitted on the data by LOESS regression model [36].

### Phenotypes

Phenotypic measurements included days to heading (DTH), days to maturity (DTM), duration of heading-to-maturity (DHTM) and plant height (PH), grain yield/m^2^ (GY) and thousand kernel weight (TKW), seed length (SEL), seed width (SEW), seed number per spike (SN), spike length (SPL) and spike weight (SPW), flag leaf length (FLL), flag leaf width (FLW), peduncle length (PL), shoot diameter (SHD) and awn length (AWL). For details on measurements and time of assessments, please refer to the manual “Physiological breeding II: a field guide to wheat phenotyping” [37].

### Data analysis

The phenotyping data of Zanjan and Alborz provinces were pooled for TDS and WW conditions separately to have a wide range of phenotypic variations in semi-arid climate zones. Then, analysis of variance (ANOVA) was conducted for the WAP under TDS and WW conditions separately using the *proc mixed* procedure in SAS version 9.4 [38]. The model for data analysis was

$$y_{ijmk}=\mu +g_{i}+l_{j}+{\left(gl\right)}_{ij}+r_{m(j)}+b_{k(mj)}+\varepsilon_{ijmk}$$

where *μ* represents the total mean, *g*_*i*_ represents the genetic effect of the *i*^*th*^ genotype, *l*_*j*_ indicates the effect of the *j*^*th*^ environment, and (*gl*)_*ij*_ indicates the interaction effect between the *i*^*th*^ genotype and the *j*^*th*^ environment. In addition, *r*_*m*(*j*)_, *b*_*k*(*mj*)_ and *ε*_*ijmk*_ represent the effect of the *m*^*th*^ replication within the *j*^*th*^ environment, the *k*^*th*^ block effect within *m*^*th*^ replication within the *j*^*th*^ environment, and the residual effect following $N(0,\sigma_{\varepsilon }^{2})$, respectively. All effects were considered as random. Heritability (*H*^2^) estimates were calculated based on each plot mean with an assumption of independence of effects using the following equation

$$H^{2}=\sigma_{g}^{2}/\left[\sigma_{g}^{2}+\sigma_{gl}^{2}/(k)+\sigma_{\varepsilon }^{2}/(rk)\right]$$

where $\sigma_{g}^{2},\;\sigma_{gl}^{2},\;\sigma_{\varepsilon }^{2},$ *k* and *r* represent genotypic variance, genotype by environment interaction variance, residual variance, the number of environments, and the number of replications, respectively. The estimation of variance components was performed by the *proc varcomp* procedure, whereas all effects were considered as random.

### GWAS

The best linear unbiased predictions (BLUPs) were estimated for each accession using the same model described for phenotypic analyzes by the lme4 package [39] in the R environment. Then, the BLUPs were used for further analysis. The mixed linear model (MLM) [40] was used for association analysis, whereas the K, K+Q, and K+PCA matrices were used in the assessments. The association analysis was separately carried out for each trait under TDS and WW conditions by GAPIT [33]. The significance threshold for MTAs was estimated with the −log_10_(*P*-value) ≥ 3.0 (*P* ≤ 0.001).

### Prediction of candidate genes

The peak markers were used to perform BLAST searches on the IWGSC v1.0 RefSeq reference genome (http://plants.ensembl.org/Triticum_aestivum/Tools/Blast). The predicted candidate genes were selected from the local LD which included the identified MTAs. The annotated genes in IWGSC v1.0, TGAC v1.0, and TAIR10 were used to predict the biological function of the candidate genes (http://plants.ensembl.org and https://triticeaetoolbox.org). The prediction of candidate genes was referred to the following criteria: a) genes identified by the peak markers, and b) genes with known biological functions for the trait under study in wheat (*T*. *aestivum*), and Arabidopsis (*A*. *thaliana*).

### GP strategy

GP was estimated by three different methods: ridge regression-best linear unbiased prediction (RR-BLUP) [41], genomic best linear unbiased prediction (GBLUP) [42], and Bayesian ridge regression (BRR) [43]. All of the GP analyses were implemented in iPat software [44]. The WAP, SBP-I, and SBP-II were assumed and assessed as three separated populations. For each population, 10%, 20%, and 33% of accessions were randomly assigned to a VS and all of the remaining accessions were used as a TS. The whole process was repeated 100 times for all of the GP methods (BRR was conducted with 10000 iterations and 1000 burn-ins as well). In addition, three marker sets (MSs) were defined to evaluate MS effect on GP accuracy. Hence, first each population was tested by its MS (WAP with 9047 SNP markers, SBP-I with 7714 SNP markers, and SBP-II with 5873 SNP markers), all of which were designated as the whole population marker set (WPMS). Then, 4785 SNP markers which were common among subpopulations were systematically separated and used to assess GEBVs. This MS was named as the common markers marker set (CMMS). The third MS included significant markers identified through GWASs with *P* ≤ 0.001, which was designated as the significant markers marker set (SMMS). The SMMS included 93 common markers identified by the K, K+Q, and K+PCA matrices for all traits and both conditions. The GP was assessed for each trait under TDS and WW conditions, separately. The accuracy of the GP was estimated as Pearson’s correlation coefficient (*r*) among GEBVs and BLUPs over TS and VS. The average of accuracies was reported across folds and repeats [45].

## Results

### Population structure and genetic relationship

**Fig 1:**
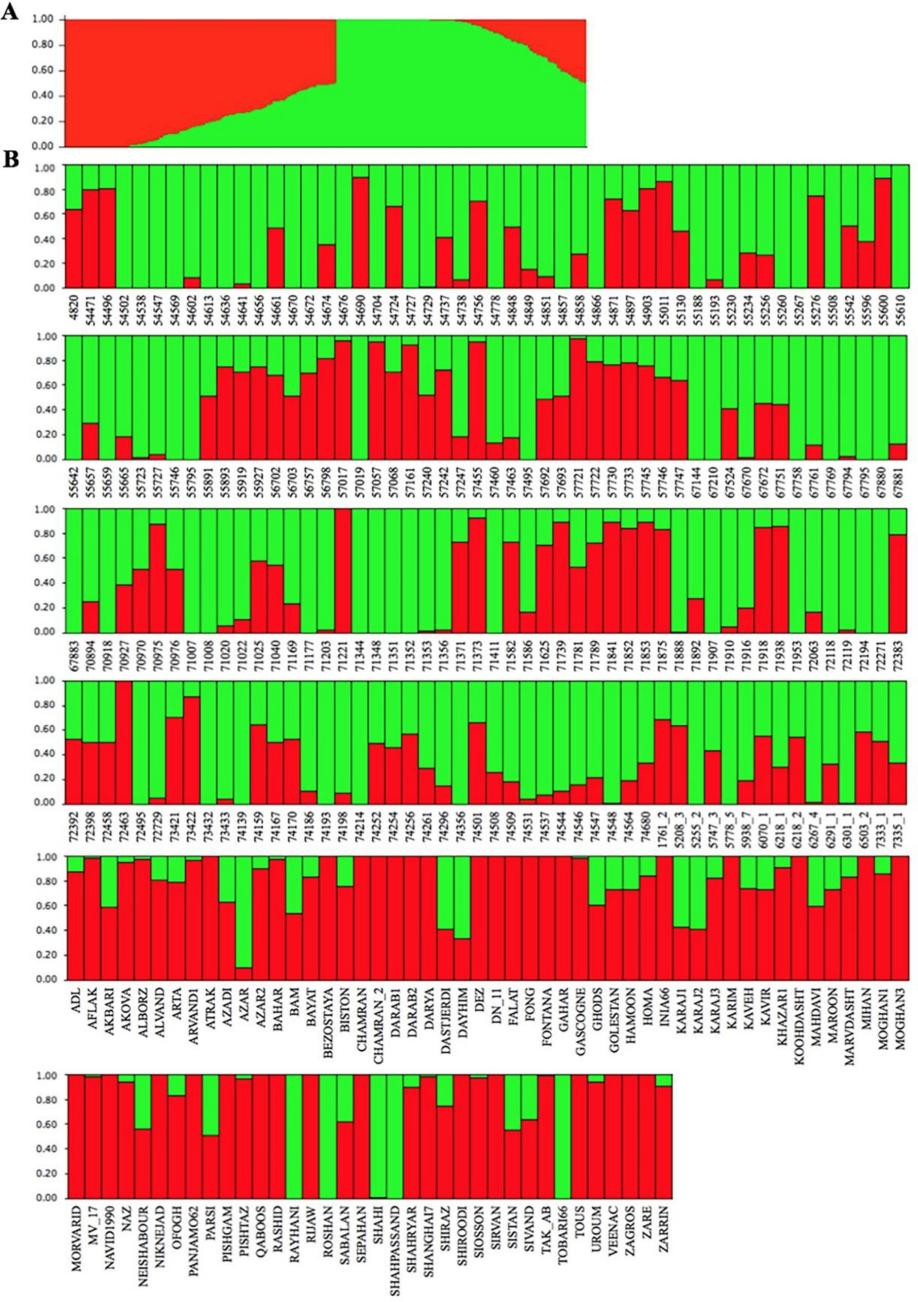
Population structure of the whole association panel using 9047 markers. (**A**) The top panel shows the population structure of 286 Iran bread wheat accessions estimated by K = 2. (**B**) The following six panels demonstrate the admixture level for each genotype. The name of each genotype is given on the x-axis. The numbers on the y-axis indicate the cluster membership coefficient (Q). The red color indicates subpopulation-I, and the green color indicates subpopulation-II.

**Fig 2:**
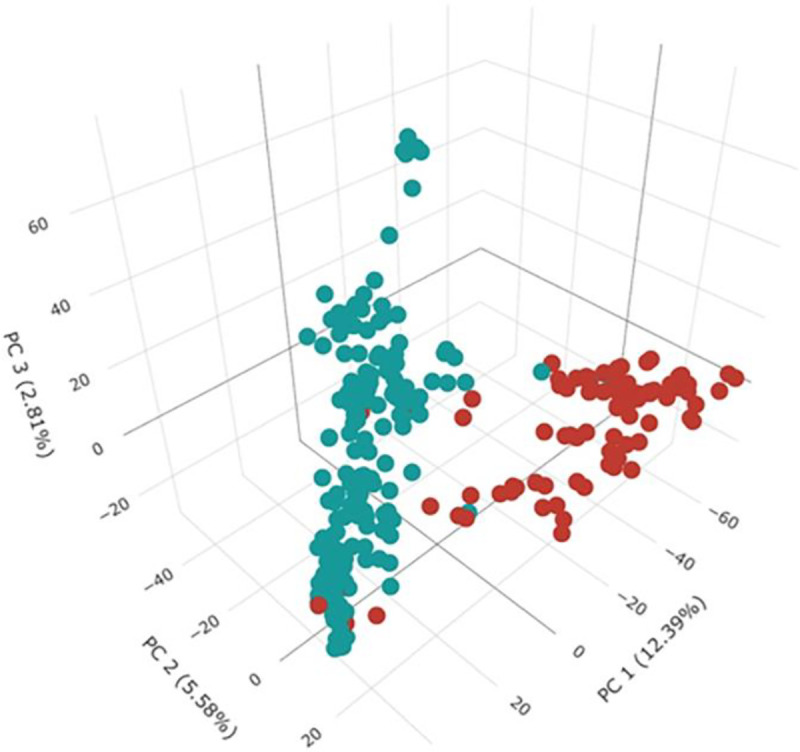
Principle component analysis (PCA) for 286 Iran bread wheat accessions using 9047 markers. The first three PCs are plotted versus each other. The cultivars and landraces are indicated with red and green colors, respectively.

The existence of two main subpopulations (Fig 1A) was identified with the use of ΔK statistic (S2 Fig in S2 File). The cluster membership coefficients (Q) showed that the SBP-I included 77 cultivars and 71 landraces, and the SBP-II including 128 landraces and ten cultivars (Fig 1B). Azar, Dastjerdi, Dayhim, Karaj1, Karaj2, Rayhani, Roshan, Shahi, Shahpassand, and Tobari66, which were introduced as cultivar, were shown high admixture level (Fig 1B). The estimated PCs for the WAP showed that PCs 1, 2, and 3 could explain 12.39, 5.58, and 2.81% of genotypic variations, respectively (Fig 2). In addition, a heat map was constructed based on the kinship values (S3 Fig in S3 File).

### Distribution of markers and LD estimates

**Fig 3:**
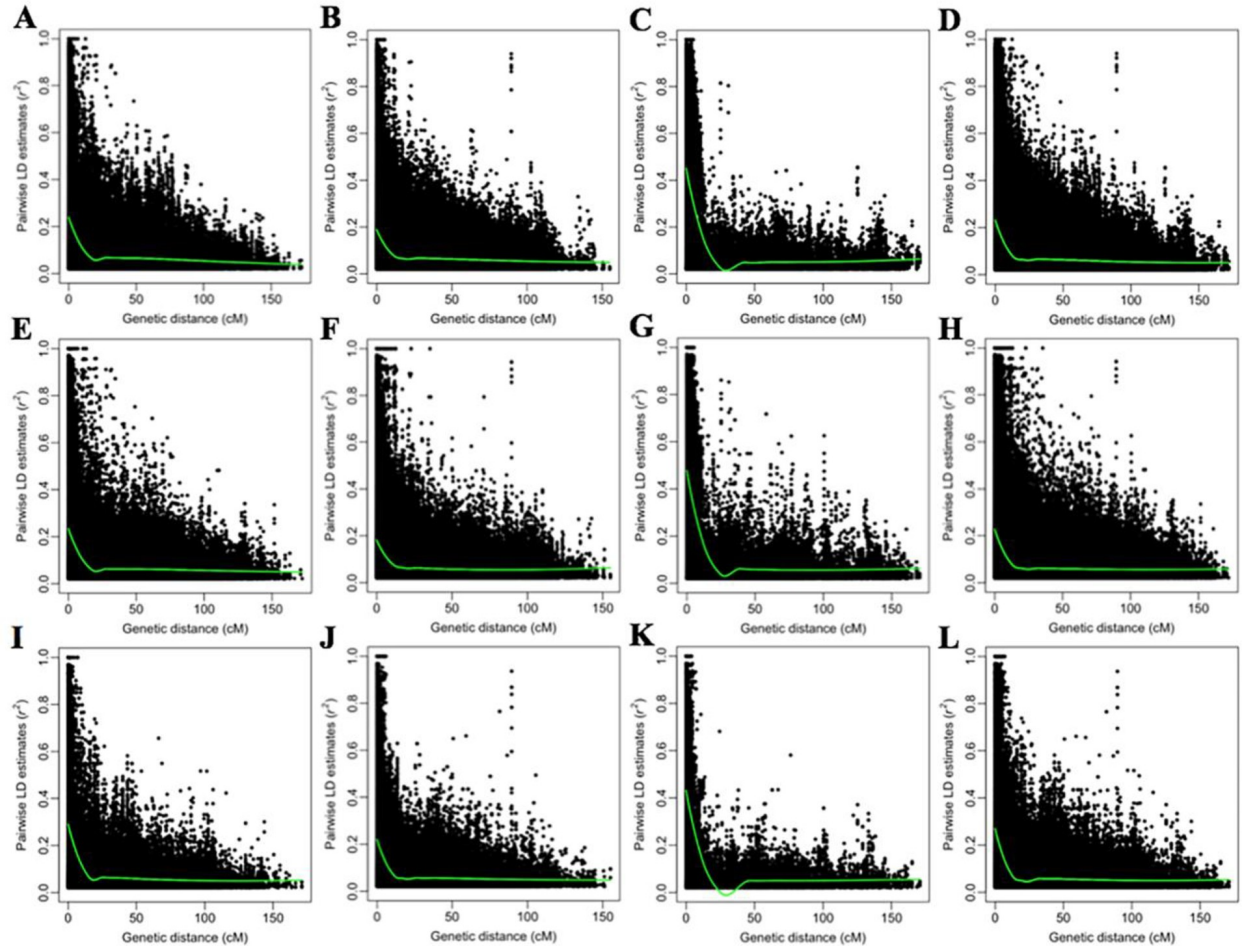
Overview of the linkage disequilibrium (LD) within the whole association panel (WAP), subpopulation-I (SBP-I), and subpopulation-II (SBP-II). The figure indicates the LD decay estimated as the squared correlation coefficient (*r*^2^) using pairwise markers plotted versus genetic distance (cM) for A genome (**A**, **E**, and **I**), B genome (**B**, **F**, and **J**), D genome (**C**, **G**, and **K**), and whole-genome (**D**, **H**, and **L**), respectively in the WAP, SBP-I, and SBP-II. The green curve lines were fitted to LD by the LOESS regression model.

A total of 9047 SNP markers were used for molecular marker analysis of the WAP. Chromosome 4D had the lowest number of markers (82), while chromosomes 2B and 3B had the highest number of markers (743 and 732, respectively) (S3 Table in S4 File). The total length of the genetic map was 2590.353 cM. The genetic map length was the shortest for chromosome 2D (85.027) but the longest for chromosome 3A (172.2) (S3 Table in S4 File). Marker density was the lowest on chromosome 4D (0.91 Marker/cM), but the highest on chromosomes 2B, 6B, and 3B (6.66, 6.23, and 6 Marker/cM, respectively) (S3 Table in S4 File). The B genome had the highest number of markers (4131), followed by A (3347) and D genomes (1569) (S3 Table in S4 File). Within WAP, LD decayed above *r*^2^ > 0.02 at about 5.24 cM in the A genome, at about 4.29 cM in the B genome, at about 9.95 cM in the D genome (Fig 3A–3C). In WAP, LD decayed above *r*^2^ > 0.02 at about 5.43 cM in the whole genome (Fig 3D). A comparison of pairwise markers with *r*^2^ > 0.02 indicated that A, B, and D genomes were contained 34.48, 57.30, and 8.22% of pairwise markers, whereas *r*^2^ means were higher in the D genome (S3 Table in S4 File). The highest percentage of pairwise markers was on chromosomes 2B (13.14%) (S3 Table in S4 File). The fewest pairwise markers were on chromosome 4D (0.23%) (S3 Table in S4 File). The distribution of molecular markers and LD estimates of the SBP-I and II are given in S4 and S5 Tables in S4 File. LD decay is demonstrated for SBP-I and II in Fig 3E–3L.

### Phenotypic data summary

ANOVA was conducted and minimum, mean, maximum, variance parameters, and heritability (*H*^2^) estimates for all traits were calculated under TDS and WW conditions, separately (S6 Table in S5 File). The phenotypic values showed less ranges under the TDS conditions for all agronomic traits, except DTH (S6 Table in S5 File). Among the accessions, significant genotype effect (*G*) was observed for all 16 agronomic traits under both TDS and WW conditions, whereas the genotype-by-environment effect (*G*×*E*) was not significant for SEL, SEW, SPL, SPW, FLL, FLW, PL, SHD, and AWL under both TDS and WW conditions (S7 and S8 Tables in S5 File). The *G*, *G*×*E*, and *H*^2^ parameters showed higher values under the WW conditions compared to the TDS conditions (S6 Table in S5 File). *H*^2^ values for agronomic traits varied from 0.47 (DTM) to 0.86 (DTH) under TDS conditions, and from 0.52 (DTM) to 0.87 (DTH) under WW conditions (S6 Table in S5 File). Pearson correlation coefficients were calculated under both conditions (S9 and S10 Tables in S5 File). DTH with DHTM (-0.66) and PH (0.61) indicated the highest correlations under the TDS conditions (S9 Table in S5 File). Furthermore, the highest correlations were observed between DTH with DHTM (-0.71) and PH (0.65) under the WW conditions (S10 Table in S5 File). DTH with DTM (0.37), DTM with DHTM (0.48), DHTM with PH (-0.45), DTH with TKW (0.32), SEW (0.30), and SPW (0.32), DTM with TKW (0.30), SEL (0.35), and SEW (-0.37), GY with TKW (0.30), and SN (0.33), TKW with SEL (0.35), SEL with SEW (0.36), and SPW (-0.31), SEW with SPW (0.34), and SN with SPW (0.30) showed more than 0.30 correlation under WW conditions (S10 Table in S5 File).

### MTAs

Association mappings were conducted using K, K+Q, and K+PCA matrices in the MLMs and utilizing 9047 SNP markers for 16 agronomic traits under TDS and WW conditions, separately. A comparison demonstrated that MLMs including K-matrix by *P* values ≤ 0.001, ≤ 0.01, and ≤ 0.05 could identify 0.06, 0.72, 4.37% of MTAs under TDS conditions and 0.06, 0.75, 4.44% of MTAs under WW conditions, respectively (S11 Table in S6 File). A combination of random effects (K-matrix) with fixed effects (Q and PCA matrices) showed that adding Q or PCA matrices to MLMs will increase the number of identified MTAs (S11 Table in S6 File). In total, 250 and 293 MTAs were identified with *P* values ≤ 0.001 for all traits under TDS and WW conditions, respectively (S12 and S13 Tables in S7 File). The highest number of MTAs was identified for AWL under TDS (36) and WW (39) conditions (S12 and S13 Tables in S7 File), being followed by DHTM under TDS (32) and WW (36) conditions (S12 and S13 Tables in S7 File). Then, the duplicated markers, the markers which were located close to each other, and markers with higher *P* values were removed, and only the peak markers were kept. Finally, 23 MTAs were selected for traits under each of the TDS and WW conditions (Table 1), whereas 17 MTAs were identified under both conditions. Such MTAs were considered as the most possible stable QTL for semi-arid environments (Table 1). Thirteen of the identified MTAs were on the A genome, fourteen of the identified MTAs on the B genome, and two of the identified MTAs on the D genome (Table 1). The highest number of the identified MTAs (4) was on chromosome 5B (Table 1). The rs65502 marker was significant among GY, SN, and SPW traits under TDS and WW conditions (Table 1). This MTA was considered as one of the most important genomic regions associated with wheat yield (Table 1). The identified MTAs encoded proteins were mostly regulator of the response to wounding, phosphorylation, protein kinase activity, hyperosmotic stress response, heat shock proteins, auxin regulation, organ development, dehydration, methylation, and transcription regulation. The predicted candidate genes and described molecular functions are provided in S14 Table in S8 File.

**Table 1:** The identified single nucleotide polymorphism (SNP) markers for 16 agronomic traits in the association panel including 286 Iran bread wheat accessions grown under terminal drought stress (TDS) and well-watered (WW) conditions in semi-arid environments, Iran.

Trait	Marker^a^	Chr^b^	Position	Alleles^c^	TDS				WW			
					Model	-log_10_ (*P* value)	MAF	Marker *R*^2^ (%)	Model	-log_10_ (*P* value)	MAF	Marker *R*^2^ (%)
DTH	rs48214	3B	68.263	(222) C/(59) T	K+Q	3.59	0.21	5.45	K+Q	3.35	0.21	5.13
DTM	rs34236	2B	62.594	(258) A/(22) T	K	3.79	0.08	6.52				
	rs28367	2A	18.219	(19) A/(262) G					K	3.69	0.07	5.46
	rs61739	4B	44.689	(28) A/(254) G					K	3.74	0.09	5.54
DHTM	rs41211	2B	72.825	(221) A/(54) G					K+PCA	4.34	0.21	8.50
	rs19295	5A	38.892	(36) A/(234) G	K	3.76	0.15	6.69	K	4.03	0.15	5.54
	rs31147	7A	71.904	(30) C/(240) G	K	3.83	0.14	6.81				
PH	rs1184	2B	55.205	(58) A/(206) G	K+PCA	3.92	0.24	6.56	K+PCA	4.25	0.24	7.31
GY	rs65502	5B	93.434	(248) A/(31) T	K+PCA	5.34	0.12	9.06	K+PCA	4.35	0.11	6.95
TKW	rs54576	2A	59.228	(53) A/(221) G	K	3.97	0.22	5.77	K	3.96	0.22	5.65
	rs17145	6A	25.146	(21) A/(252) G	K+Q	3.49	0.10	4.98	K	3.96	0.09	5.65
SEL	rs998	6A	53.619	(154) A/(108) G	K+Q	4.91	0.42	7.18	K+Q	5.38	0.41	7.70
SEW	rs55852	3B	77.361	(139) A/(123) C	K	3.63	0.45	4.88	K	3.78	0.45	5.13
SN	rs51365	2D	13.642	(222) A/(52) C	K+PCA	3.61	0.20	6.11				
	rs65502	5B	93.434	(248) A/(31) T	K+Q	4.54	0.12	6.14	K+Q	4.06	0.11	5.44
	rs64054	7A	58.263	(207) A/(63) G	K	3.5	0.23	4.68				
SPL	rs60932	3A	53.669	(24) A/(254) C	K+PCA	5.03	0.10	7.59	K+PCA	5.01	0.09	7.51
SPW	rs15276	1A	44.512	(204) C/(69) T					K+Q	3.55	0.26	5.32
	rs2368	1B	32.984	(218) G/(44) T					K+Q	3.45	0.20	5.18
	rs65502	5B	93.434	(248) A/(31) T	K+PCA	4.09	0.12	8.99				
	rs53982	5D	132.023	(249) C/(33) T	K+PCA	3.96	0.10	8.79	K+PCA	3.29	0.10	7.94
FLL	rs58293	4B	50.376	(264) C/(19) G					K+Q	3.47	0.07	4.69
	rs59732	6B	60.336	(234) C/(38) T	K+PCA	3.79	0.16	5.36	K+PCA	3.79	0.16	4.97
FLW	rs6770	3B	45.525	(19) C/(245) T	K+Q	3.95	0.10	6.02				
PL	rs31423	7A	32.091	(207) A/(60) G	K+PCA	3.28	0.23	5.45	K+PCA	3.32	0.23	5.07
SHD	rs11489	4A	136.19	(265) C/(14) T	K+PCA	4.05	0.05	6.84	K+PCA	3.87	0.05	6.64
	rs49193	6A	25.146	(258) G/(21) T	K	4.15	0.08	5.81	K	4.12	0.07	5.81
AWL	rs59275	2A	59.228	(95) A/(170) G	K+PCA	4.71	0.37	6.73	K+PCA	4.08	0.35	5.63
	rs8958	5B	35.359	(235) C/(35) T	K+PCA	3.69	0.14	5.06	K+PCA	4.19	0.14	5.95

The number of homozygous alleles is given in parentheses. The type of statistical matrices used in the GWASs is also provided.

### GP

**Fig 4:**
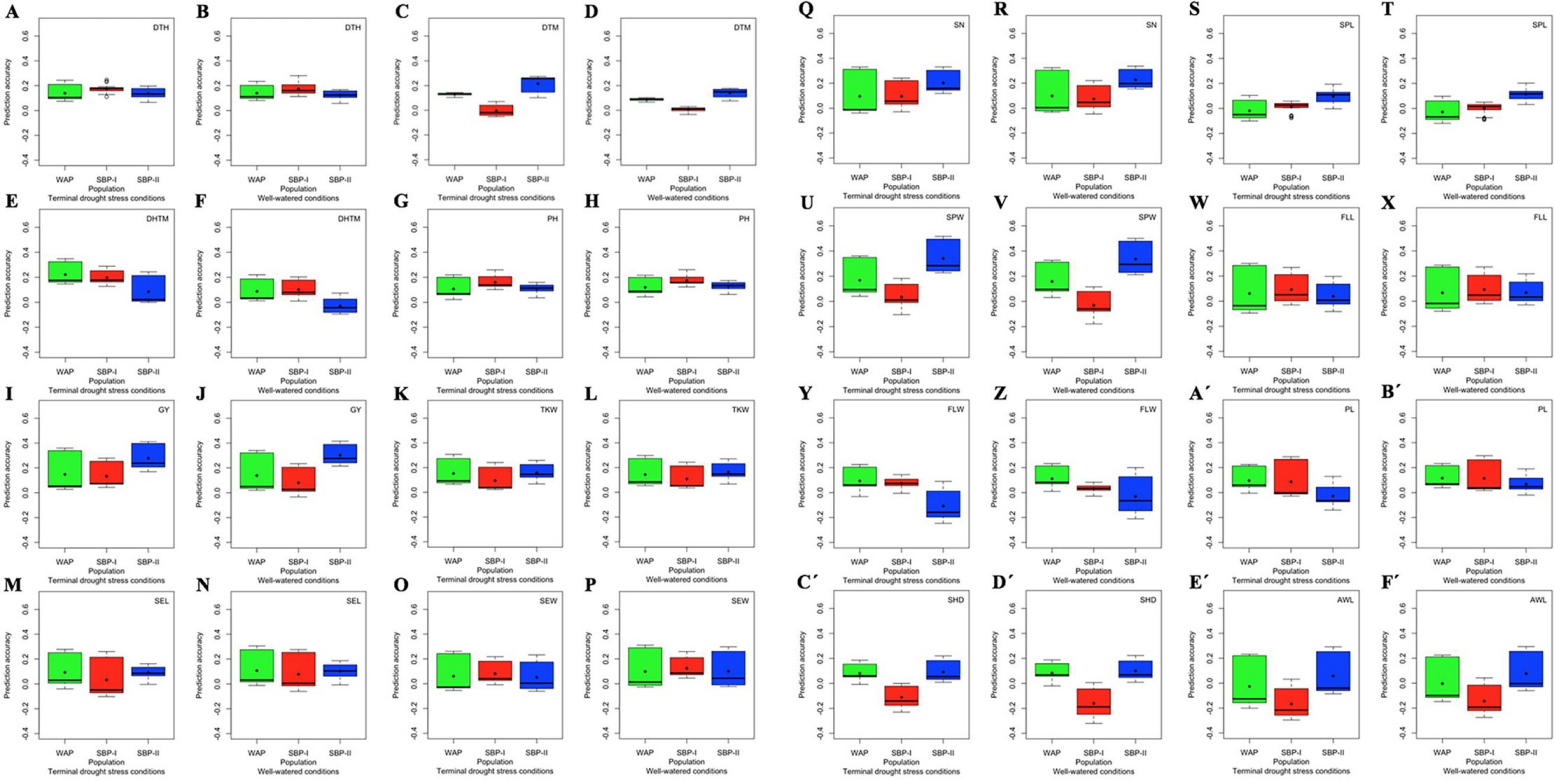
The effect of population on genomic prediction (GP) accuracy for 16 wheat agronomic traits under terminal drought stress (TDS) and well-watered (WW) conditions. **A**-**F´)** The accuracy of GP in the whole association panel (WAP), subpopulation-I (SBP-I), and subpopulation-II (SBP-II) are shown with green, red, and blue colors, respectively. The prediction accuracies were calculated by ridge regression-best linear unbiased prediction (RR-BLUP), genomic best linear unbiased predictions (GBLUP), and Bayesian ridge regression (BRR) methods. The boxplots show the first, second (median) and third quartile. The middle points indicate a mean of GP accuracies for the trait of interest. DTH, days to heading; DTM, days to maturity; DHTM, duration of heading-to-maturity; PH, plant height (cm); GY, grain yield (kg/m^2^); TKW, thousand kernel weight (g); SEL, seed length (mm); SEW, seed width (mm); SN, seed number per spike (number); SPL, spike length (cm); SPW, spike weight (g); FLL, flag leaf length (cm); FLW, flag leaf width (mm); PL, peduncle length (cm); SHD, shoot diameter (mm) and AWL, awn length (cm).

The prediction accuracies varied from -0.32 to 0.52 (S15 Table in S9 File). Three traits in SBP-I, seven traits in SBP-II, and six traits in WAP showed the highest prediction accuracies under both TDS and WW conditions (S15 Table in S9 File). The DHTM (0.35 and 0.22), TKW (0.31 and 0.30), SEL (0.28 and 0.31), SEW (0.26 and 0.31), FLL (0.30 and 0.29) and FLW (0.22 and 0.23) in WAP, DTH (0.25 and 0.28), PH (0.26 and 0.26) and PL (0.29 and 0.30) in SBP-I, and DTM (0.27 and 0.18), GY (0.41 and 0.42), SN (0.33 and 0.34), SPL (0.19 and 0.20), SPW (0.52 and 0.50), SHD (0.22 and 0.22) and AWL (0.29 and 0.29) in SBP-II, showed the highest prediction accuracies under TDS and WW conditions, respectively (S15 Table in S9 File, Fig 4).

**Fig 5:**
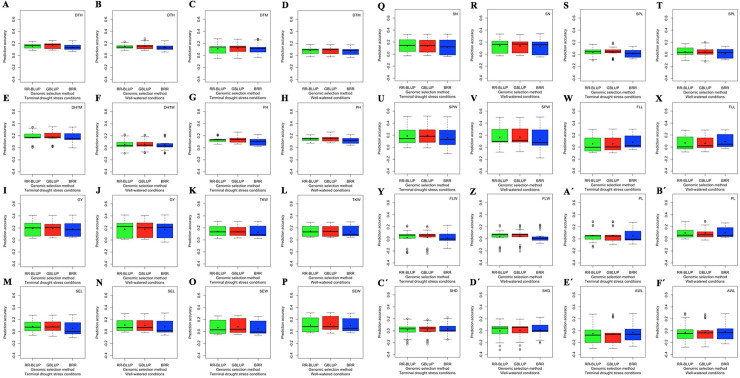
The effect of genomic selection (GS) method on genomic prediction (GP) accuracy for 16 wheat agronomic traits under terminal drought stress (TDS) and well-watered (WW) conditions. **A**-**F´)** The accuracy of GP for ridge regression-best linear unbiased prediction (RR-BLUP), genomic best linear unbiased predictions (GBLUP), and Bayesian ridge regression (BRR) genomic selection (GS) methods are demonstrated with green, red, and blue colors, respectively. The GP accuracies were calculated across the whole association panel (WAP), subpopulation-I (SBP-I), and subpopulation-II (SBP-II). The boxplots show the first, second (median) and third quartile. The middle points indicate a mean of GP accuracies for the trait of interest. DTH, days to heading; DTM, days to maturity; DHTM, duration of heading-to-maturity; PH, plant height (cm); GY, grain yield (kg/m^2^); TKW, thousand kernel weight (g); SEL, seed length (mm); SEW, seed width (mm); SN, seed number per spike (number); SPL, spike length (cm); SPW, spike weight (g); FLL, flag leaf length (cm); FLW, flag leaf width (mm); PL, peduncle length (cm); SHD, shoot diameter (mm) and AWL, awn length (cm).

The RR-BLUP, GBLUP, and BRR methods identified the highest prediction accuracies for 2, 8, and 6 phenotypes under TSD conditions, and 3, 5, and 8 phenotypes under WW conditions, respectively (S15 Table in S9 File). The highest prediction accuracies were identified by the GBLUP method for DTH, DHTM, PH, SEW, SPL, SPW, FLL, and PL, by the RR-BLUP method for DTM and TKW, and by the BRR method for GY, SEL, SN, FLW, SHD, and AWL under TDS conditions (S15 Table in S9 File, Fig 5). Likewise, the highest prediction accuracies were identified by the GBLUP method for DTH, PH, SEW, SPL, and PL, by the RR-BLUP method for DTM, DHTM, and AWL, and by the BRR method for GY, TKW, SEL, SN, SPW, FLL, FLW, and SHD under WW conditions (S15 Table in S9 File, Fig 5). It was notable that none of the estimated highest prediction accuracies were identified by the RR-BLUP and BRR methods in the SBP-I (S15 Table in S9 File).

**Fig 6:**
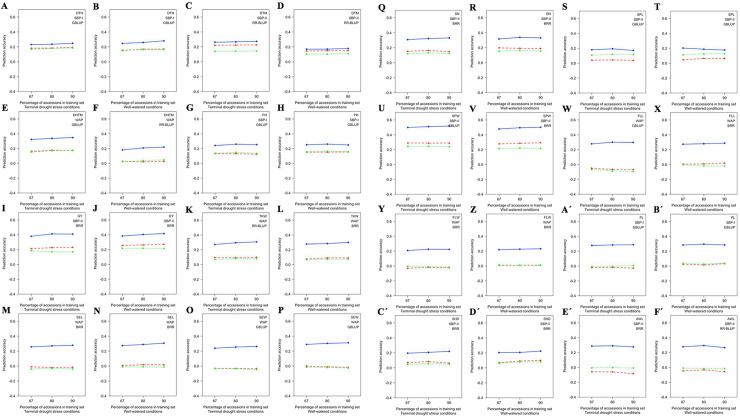
The effect of training set (TS) size on genomic prediction (GP) accuracy for 16 wheat agronomic traits under terminal drought stress (TDS) and well-watered (WW) conditions. **A-F´)** The GP accuracies are provided after selecting population and genomic selection (GS) method. The TSs were included 67, 80, and 90 percentage of genotypes of each population. Three marker sets (MSs) were used during the assessments. The whole population marker set (WPMS) included 9047, 7714, and 5873 markers for WAP, SBP-I, and SBP-II, respectively, the common markers marker set (CMMS) included 4785 common markers among subpopulations, and the significant markers marker set (SMMS) included 93 significant markers identified through GWASs which were demonstrated by dotted, dashed, and solid lines with green, red, and blue colors, respectively. DTH, days to heading; DTM, days to maturity; DHTM, duration of heading-to-maturity; PH, plant height (cm); GY, grain yield (kg/m^2^); TKW, thousand kernel weight (g); SEL, seed length (mm); SEW, seed width (mm); SN, seed number per spike (number); SPL, spike length (cm); SPW, spike weight (g); FLL, flag leaf length (cm); FLW, flag leaf width (mm); PL, peduncle length (cm); SHD, shoot diameter (mm) and AWL, awn length (cm).

The TS and MS effects were identified for each phenotype after classifying phenotypes by the selected population and the GS method to attain the highest GP accuracies. The highest prediction accuracies were identified for ten phenotypes (DTH, DTM, DHTM, TKW, SEL, SEW, SN, SPW, PL, and SHD) under TDS conditions and eleven phenotypes (DTH, DTM, DHTM, GY, TKW, SEL, SEW, SPW, FLL, FLW, and SHD) under WW conditions when 90% of accessions applied in TS (S15 Table in S9 File). Therefore, no sign of reaching the plateau of the prediction accuracy was observed for these phenotypes (Fig 6). PH and AWL showed the highest prediction accuracy under both TDS and WW conditions when 80% of the population applied in TS (Fig 6). GY, SPL, FLL, and FLW under TDS conditions, and SN and PL under WW conditions indicated the highest prediction accuracy when 80% of the population applied in TS (S15 Table in S9 File). Consequently, a sign of reaching the plateau of the prediction accuracy was seen for these phenotypes (Fig 6). In addition, the highest accuracy of the GP was seen for SPL under the WW conditions, when 67% of the population applied in TS (Fig 6).

**Fig 7:**
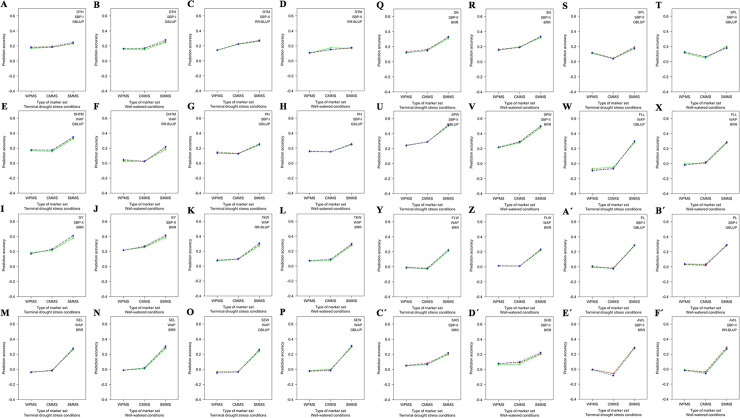
The effect of type of marker set (MS) on genomic prediction (GP) accuracy for 16 wheat agronomic traits under terminal drought stress (TDS) and well-watered (WW) conditions. **A-F´)** The GP accuracies are provided after selecting population and genomic selection (GS) method. Three MSs were used during the assessments. The whole population marker set (WPMS) included 9047, 7714, and 5873 markers for WAP, SBP-I, and SBP-II, respectively, the common markers marker set (CMMS) included 4785 common markers among subpopulations, and the significant markers marker set (SMMS) included 93 significant markers identified through GWASs. The TSs were included 67, 80, and 90 percentage of genotypes of each population which were demonstrated by solid, dashed, and doted lines, with green, red, and blue colors, respectively. DTH, days to heading; DTM, days to maturity; DHTM, duration of heading-to-maturity; PH, plant height (cm); GY, grain yield (kg/m^2^); TKW, thousand kernel weight (g); SEL, seed length (mm); SEW, seed width (mm); SN, seed number per spike (number); SPL, spike length (cm); SPW, spike weight (g); FLL, flag leaf length (cm); FLW, flag leaf width (mm); PL, peduncle length (cm); SHD, shoot diameter (mm) and AWL, awn length (cm).

The SMMS (93 significant markers) showed the highest prediction accuracy for all traits under both conditions (S15 Table in S9 File, Fig 7). The SMMS, also, was produced higher prediction accuracies in all of the GS methods, compared to the CMMS and WPMS (S15 Table in S9 File, Fig 7).

## Discussion

### Effect of population on GP accuracy

The cross-validation (CV) results revealed differences in the prediction accuracies which were only explainable by population structure. If we assume the SBP-I as a half-pure population (77 cultivars and 71 landraces), the SBP-II as a diverse population (128 landraces and 10 cultivars), and the WAP as a mixed population (87 cultivars and 199 landraces), it is expected that a mixed population will perform more reliably because of additional diversity in TS and more cultivars in VS during CV repeats [18, 19]. A mixed population may have an adequate size in a breeding program, while the main issue is whether it contains more diverse or breeding genotypes. The best strategy would be using a large TS with high diversity which can be compared with either diverse or breeding VS. However, a breeding VS will provide more exact results for the trait of interest [19]. This strategy will ensure that no genotype has a full relationship in TS and VS, and therefore the possibility of obtaining reliable results would be more [15, 21]. It was reported that the prediction accuracy was better when two less-related groups of genotypes were combined [19]. A randomized combination of accessions from the subpopulations showed higher prediction accuracy compared to predictions within the subpopulations [16]. In the present study, the highest prediction accuracies for DHTM, TKW, SEL, SEW, FLL, and FLW was seen in WAP, indicating that TS has suitable size and diversity for these traits. In SBP-I, it is not clear that the estimated prediction accuracies for DTH, PH, and PL are because of the TS size or diversity since the population included 52% cultivar and 48% landrace. This study hypothesized that probably older genotypes could preserve information related to predict the performance of new genotypes in SBP-I. The SBP-II was shaped from about 92% landrace. Therefore, the identified prediction accuracies for DTM, GY, SN, SPL, SPW, SHD, and AWL have relied more on the diversity of TS rather than the size of TS.

### Effect of GS method on GP accuracy

It was reported that RR-BLUP works well for genetic architectures containing many loci with small effects [46]. If heritability is stable, the RR-BLUP will not be sensitive to the genetic architecture of the trait [46]. The Bayesian methods will improve prediction accuracy when the number of QTL decreases and effects increase [47]. However, RR-BLUP and GBLUP are mathematically equivalent [14], if a population is an advanced-breeding population and markers are closely related to the trait of interest, more genetic variance will be achieved by the GBLUP compared to other GS methods [48]. RR-BLUP assumes all markers with a similar variance [14] and shrinks marker effects equally toward zero. GBLUP derives genetic relationships from predictors and estimates breeding values from the relationship matrix using a BLUP model [49]. BRR is similar to RR-BLUP and shrinks all effects of the markers toward zero [50]. However, the shrinkage depends on the population size [51]. In the present study, the highest prediction accuracies were identified in the SBP-I only by the GBLUP method. Hence, this study concluded that the GBLUP detected genetic effects better in this population due to the presence of more cultivars in the SBP-I. The RR-BLUP method had better performance in the WAP and SBP-II, which were mixed and diverse populations, respectively. Therefore, this study concluded that probably the RR-BLUP could identify loci with minor genetic effects in the WAP and SBP-II. The results of BRR method was similar to the RR-BLUP method and identified linkages among markers and QTL better in the WAP and SBP-II. It was reported that, the performance of the population with multi-subpopulations is dependent on the existence of ancestral LD which is common across subpopulations [52]. The GS methods which can obtain marker-QTL LD would have more effective outcomes compared to methods which take the genetic relationships between training and validation populations into the model [53, 54]. The RR-BLUP and BRR are the linear models which assume the linearity of marker effects. With having perfect linkages among markers and QTL, a larger population would increase GP accuracy [47, 55]. Optimizing TS and removing less related markers can improve prediction accuracy if the linkages among markers and QTL are not perfect [48, 55].

### Effect of TS on GP accuracy

This study assumed that probably some closely related accessions were present in the association panel. If the related individuals are present in both TS and VS, the inflation produced by half and full-sib families may lead to false results during the CV repeats. Differences among prediction accuracies decrease in larger TSs [19]. The Bayesian Cπ outperformed RR-BLUP for four out of five phenotypes when 90 to 100 lines were in TS [19]. Bayesian B showed similar prediction accuracy with GBLUP in a large TS [56]. In this study, the WAP and SBP-II showed that eight phenotypes (DTM, DHTM, TKW, SEL, SEW, SN, SPW, and SHD) under TDS conditions and ten phenotypes (DTM, DHTM, GY, TKW, SEL, SEW, SPW, FLL, FLW, and SHD) under WW conditions have had the highest prediction accuracy with the use of 90% of the population in TS. This study concluded that a high level of diversity in TS led to the highest prediction accuracy for phenotypes identified in the mixed (WAP) and diverse (SBP-II) populations. Such results are in line with many types of research [56, 57]. The highest prediction accuracy observed for GY, SPL, FLL, FLW, and AWL, under TDS, and for SN, SPL, and AWL, under WW conditions, when 80% of accessions utilized in the TSs of the WAP and SBP-II. These results may indicate that less diversity was needed to evaluate the prediction accuracy of these phenotypes. Developing a robust LD across generations is necessary for preserving prediction accuracy since previous generations probably will have less relationship to new generations [58]. When 80 to 90% of SBP-I was utilized in the TS under both conditions, the plateau of the prediction accuracy was seen for DTH, PH, and PL. Since the SBP-I was shaped from 48% landrace and 52% cultivar, it is not clear that the highest prediction accuracies for DTH, PH, and PL are due to diversity or inbred genotypes in the TS. The only trait showed the highest prediction accuracy by 67% of accessions in TS was SPL in the SBP-II under the WW conditions. The most likely explanation is that TS and VS have been fairly diverse for SPL, so that TS could evaluate VS very well.

### Effect of MS on GP accuracy

The ability of GS to enhance plant breeding is based on the fact that genotyping will soon become cheaper, and consequently, breeders will be able to save time and reduce phenotyping tasks [8, 15]. Although, higher prediction accuracy will be obtained with increasing marker density [56, 59], if the marke set is in a direct linkage with the traits of interest, GS would potentially explain all genotypic variables which are in LD [60]. Therefore, not only appropriate marker density should be identified, markers that are in LD should be used for attaining the highest prediction accuracy which may reduce the costs of the breeding programs [16, 61]. This study integrated the output of GWASs with different GS methods. The results revealed that all of the markers used in the present study were not necessary to achieve the highest attainable GP accuracy. The prediction accuracies were slightly increased or remained constant for all traits (except SPL, PL, and AWL) with the use of CMMS since the uncommon markers between subpopulations were deleted from the GS methods. The GP accuracies increased for all traits using SMMS. It is believed that GP with a reduced number of significant markers may have the same shortcomings as the marker-assisted recurrent selection method [62]. The results of the present study suggest further investigations to avoid future challenges.

## Conclusion

This study concluded that obtaining the highest GP accuracy depends on the extent of LD, the genetic architecture of trait, genetic diversity of the population, and the GS method.

## Supporting information

S1 File S1 and S2 Tables are lists of the 199 landraces and 87 cultivars from Iran bread wheat germplasm used in the present study. (XLSX)

S2 File S1 Fig shows climate conditions in fields during the 2017–2018 cropping season, and S2 Fig demonstrates ΔK values for population structure. (DOCX)

S3 File S3 Fig provides a heat map of the kinship values for 286 Iran bread wheat accessions used in the present study. (PDF)

S4 File S3-S5 Tables are provided information about the distribution of molecular markers and linkage disequilibrium estimates in the whole association panel and subpopulations. (DOCX)

S5 File S6 Table is provided information about descriptive statistics and variance parameters, S7 and S8 Tables are the results of the analysis of variance, and S9 and S10 Tables are the Pearson correlation coefficients for 16 agronomic traits under terminal drought stress and well-watered conditions. (DOCX)

S6 File S11 Table compares the statistical power of K, K+Q, and K+PCA matrices for genome-wide association mapping, in the present study. (DOCX)

S7 File S12 and S13 Tables are lists of single nucleotide polymorphisms identified through genome-wide association studies for 16 agronomic traits under terminal drought stress and well-watered conditions, respectively. (XLSX)

S8 File S14 Table describes selected markers for 16 agronomic traits. (DOCX)

S9 File S15 Table provides genomic prediction accuracy for 16 agronomic traits under terminal drought stress and well-watered conditions as well as a summary. (XLSX)
