# Supplementary material for: A classic approach for determining genomic prediction accuracy under terminal drought stress and well-watered conditions in wheat landraces and cultivars
Source: PLoS One. 2021 Mar 5;16(3):e0247824. doi: 10.1371/journal.pone.0247824 (PMC7935232; doi:10.1371/journal.pone.0247824)
Supplement: S2 File — (DOCX) [file pone.0247824.s002.docx]

Fig. S1 Climate conditions including average temperature (^o^C) and precipitation (mm) (**A**) at the Agricultural Research Farm of Karaj Islamic Azad University, and (**B**) the Kheirabad Agricultural Research Station during the 2017-2018 cropping season.


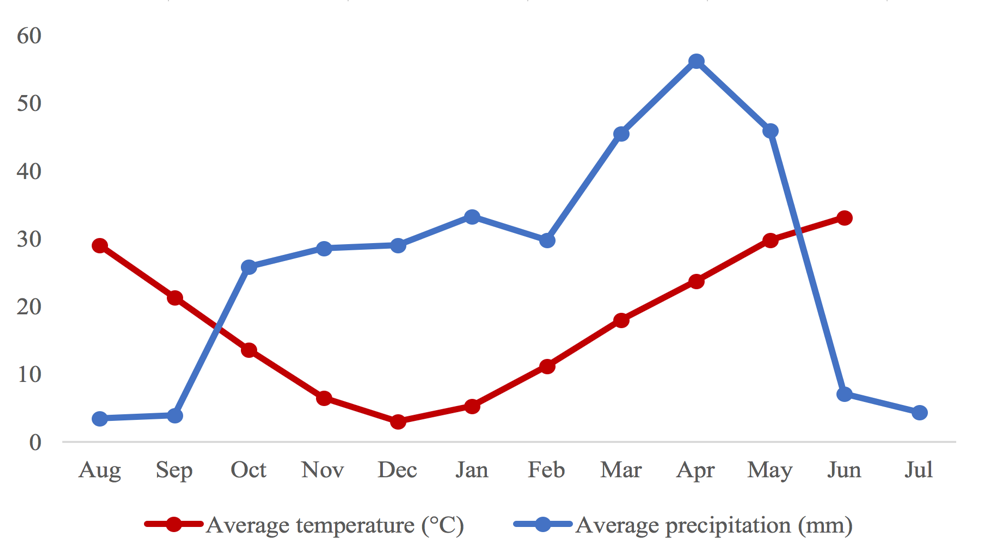

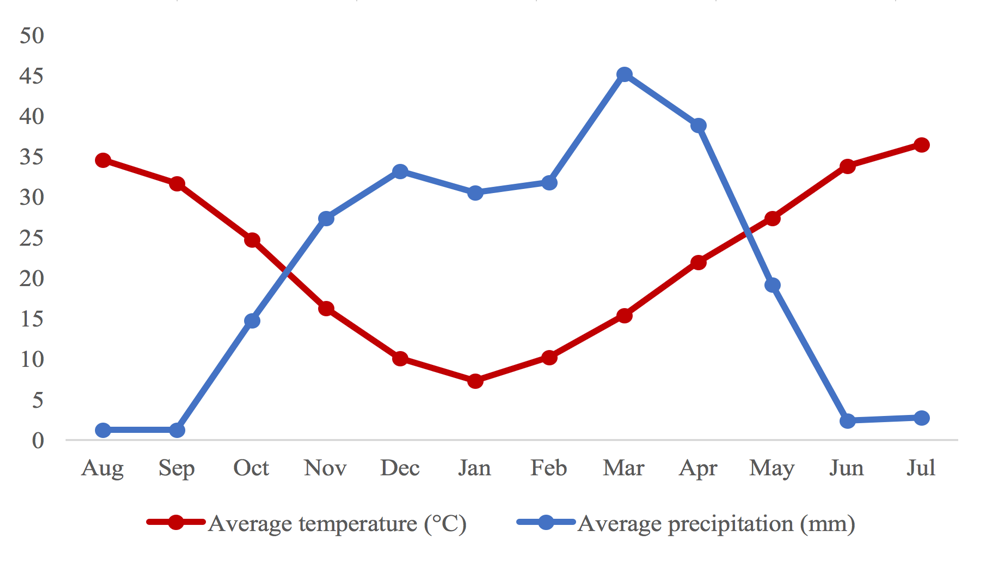


**A**

**B**


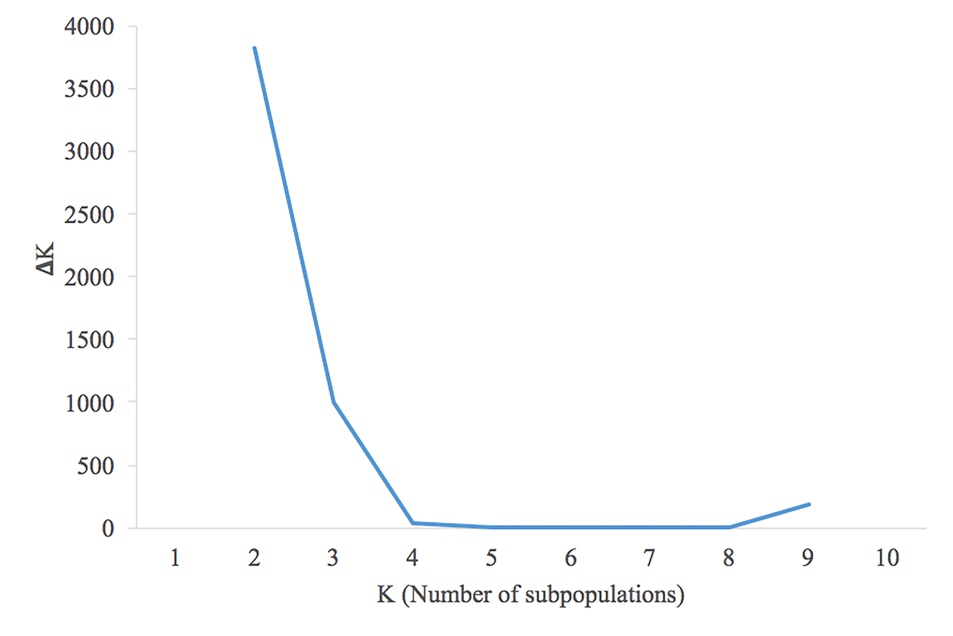


Fig. S2. ∆K values calculated for K = 1 to 10 to determine the number of subpopulations in the association panel including 286 Iran bread wheat accessions.
