# Supplementary figures and images for: A classic approach for determining genomic prediction accuracy under terminal drought stress and well-watered conditions in wheat landraces and cultivars
Source: PLoS One. 2021 Mar 5;16(3):e0247824. doi: 10.1371/journal.pone.0247824 (PMC7935232; doi:10.1371/journal.pone.0247824)

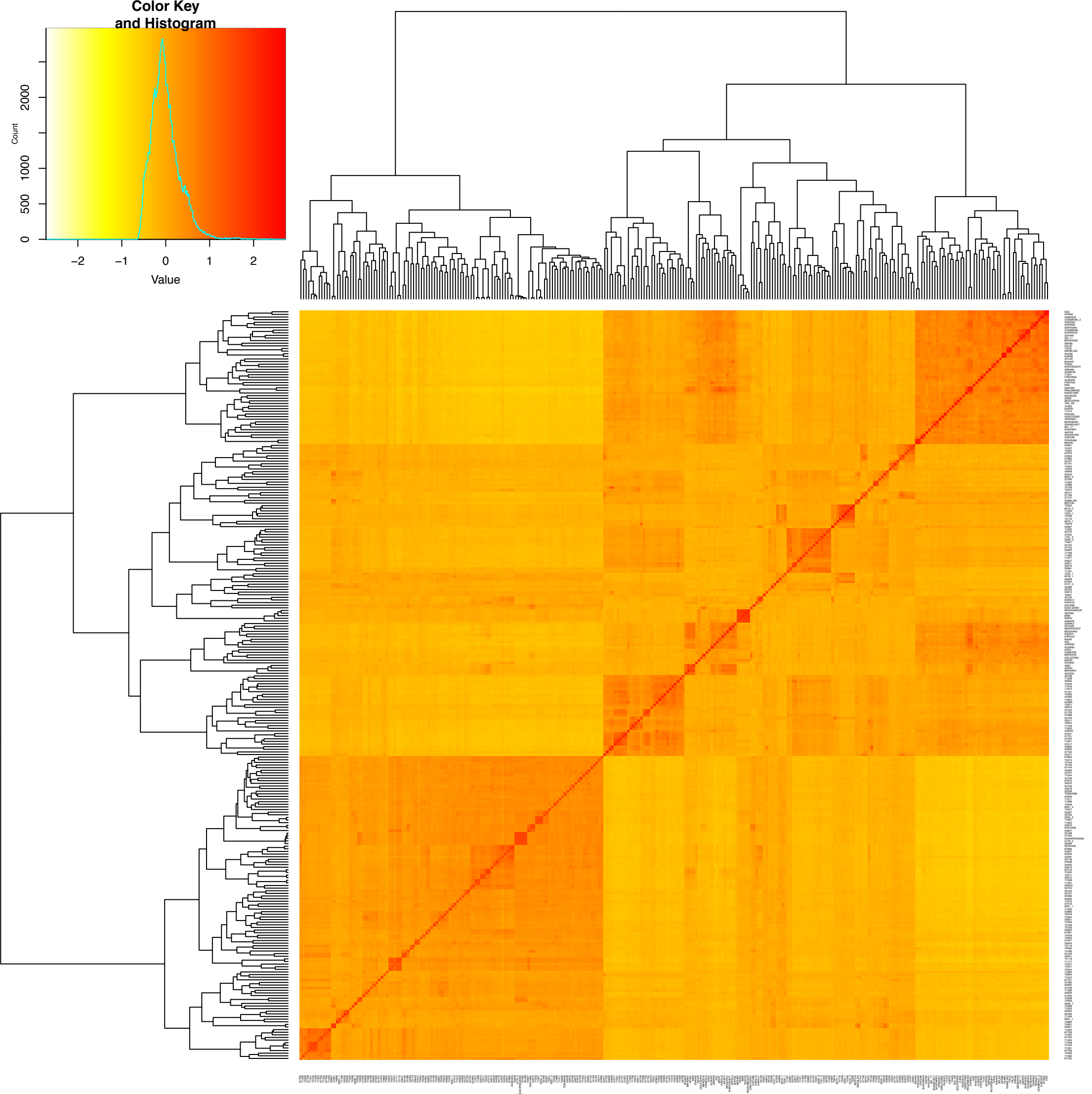

Fig. S3 Heat map of the kinship values for 286 Iran bread wheat accessions.

Supplement: S3 File — (PDF) [file pone.0247824.s003.pdf]
