# Supplementary material for: A classic approach for determining genomic prediction accuracy under terminal drought stress and well-watered conditions in wheat landraces and cultivars
Source: PLoS One. 2021 Mar 5;16(3):e0247824. doi: 10.1371/journal.pone.0247824 (PMC7935232; doi:10.1371/journal.pone.0247824)
Supplement: S4 File — (DOCX) [file pone.0247824.s004.docx]

Table S3. Distribution of molecular markers and linkage disequilibrium (LD) estimates in the association panel including 199 landraces and 87 cultivars from Iran bread wheat germplasm.

| Genome | Chr^a^ | Markers distribution | | |  | LD estimates | | |
| --- | --- | --- | --- | --- | --- | --- | --- | --- |
|  |  | Number  of markers | Genetic map  length (cM) | Marker density (Marker/cM) |  | Pairwise markers  ($r^{2}>$ 0.02) | $r^{2}$ mean ($r^{2}>$ 0.02) | LD ($r^{2}>$ 0.02) |
| A genome | 1A | 499 | 117.878 | 4.23 |  | 45429 (5.09%) | 0.10 | 4.59 |
|  | 2A | 527 | 92.517 | 5.7 |  | 60157 (6.74%) | 0.12 | 5.68 |
|  | 3A | 492 | 172.2 | 2.86 |  | 43061 (4.82%) | 0.09 | 9.28 |
|  | 4A | 397 | 152.121 | 2.61 |  | 25073 (2.81%) | 0.11 | 5.12 |
|  | 5A | 398 | 111.967 | 3.55 |  | 27370 (3.07%) | 0.09 | 4.66 |
|  | 6A | 385 | 99.391 | 3.87 |  | 27586 (3.09%) | 0.10 | 5.71 |
|  | 7A | 649 | 135.625 | 4.79 |  | 79138 (8.87%) | 0.10 | 4.73 |
|  | Total | 3347 | 881.699 | 3.8 |  | 307814 (34.48%) | 0.10 | 5.24 |
| B genome | 1B | 584 | 113.814 | 5.13 |  | 65780 (7.37%) | 0.10 | 3.45 |
|  | 2B | 743 | 111.506 | 6.66 |  | 117256 (13.14%) | 0.09 | 5.13 |
|  | 3B | 732 | 121.909 | 6 |  | 108123 (12.11%) | 0.09 | 6.25 |
|  | 4B | 243 | 102.696 | 2.37 |  | 10044 (1.13%) | 0.11 | 5.42 |
|  | 5B | 635 | 155.004 | 4.1 |  | 78448 (8.79%) | 0.09 | 6.25 |
|  | 6B | 610 | 97.872 | 6.23 |  | 74711 (8.37%) | 0.09 | 2.84 |
|  | 7B | 584 | 118.551 | 4.93 |  | 57104 (6.40%) | 0.09 | 2.84 |
|  | Total | 4131 | 821.352 | 5.03 |  | 511466 (57.30%) | 0.09 | 4.29 |
| D genome | 1D | 295 | 136.487 | 2.16 |  | 15213 (1.70%) | 0.14 | 6.89 |
|  | 2D | 375 | 85.027 | 4.41 |  | 27237 (3.05%) | 0.22 | 10.24 |
|  | 3D | 187 | 126.448 | 1.48 |  | 5179 (0.58%) | 0.15 | 4.55 |
|  | 4D | 82 | 90.119 | 0.91 |  | 2032 (0.23%) | 0.18 | 6.83 |
|  | 5D | 176 | 170.702 | 1.03 |  | 5265 (0.59%) | 0.13 | 3.42 |
|  | 6D | 197 | 121.074 | 1.63 |  | 7394 (0.83%) | 0.11 | 5.70 |
|  | 7D | 257 | 157.445 | 1.63 |  | 11029 (1.24%) | 0.14 | 12.62 |
|  | Total | 1569 | 887.302 | 1.77 |  | 73349 (8.22%) | 0.17 | 9.95 |
| Whole genome | | 9047 | 2590.353 | 3.49 |  | 892629 | 0.10 | 5.43 |

^a^Chromosome; $r^{2}$is the squared correlation coefficient of the pairwise markers.

Table S4. Distribution of molecular markers in the subpopulation-I (SBP-I) and subpopulation-II (SBP-II).

| Genome | Chr^a^ | SBP-I | | |  | SBP-II | | |
| --- | --- | --- | --- | --- | --- | --- | --- | --- |
|  |  | Number  of markers | Genetic map  length (cM) | Marker density (Marker/cM) |  | Number  of markers | Genetic map  length (cM) | Marker density (Marker/cM) |
| A genome | 1A | 441 | 117.878 | 3.74 |  | 306 | 117.878 | 2.59 |
|  | 2A | 459 | 92.517 | 4.96 |  | 374 | 92.517 | 4.04 |
|  | 3A | 428 | 172.2 | 2.49 |  | 271 | 171.063 | 1.58 |
|  | 4A | 327 | 152.121 | 2.15 |  | 253 | 152.121 | 1.66 |
|  | 5A | 354 | 111.967 | 3.16 |  | 222 | 111.967 | 1.98 |
|  | 6A | 344 | 99.391 | 3.46 |  | 282 | 99.391 | 2.83 |
|  | 7A | 541 | 135.625 | 3.99 |  | 454 | 135.635 | 3.34 |
|  | Total | 2894 | 881.699 | 3.28 |  | 2162 | 880.572 | 2.45 |
| B genome | 1B | 484 | 113.814 | 4.25 |  | 377 | 113.814 | 3.31 |
|  | 2B | 652 | 111.506 | 5.85 |  | 484 | 111.506 | 4.34 |
|  | 3B | 610 | 121.909 | 5.00 |  | 457 | 121.909 | 3.74 |
|  | 4B | 204 | 102.696 | 1.99 |  | 146 | 102.696 | 1.42 |
|  | 5B | 581 | 155.004 | 3.75 |  | 390 | 155.004 | 2.51 |
|  | 6B | 506 | 97.872 | 5.17 |  | 388 | 97.872 | 3.96 |
|  | 7B | 515 | 118.551 | 4.34 |  | 410 | 118.551 | 3.45 |
|  | Total | 3552 | 821.352 | 4.32 |  | 2652 | 821.352 | 3.23 |
| D genome | 1D | 235 | 136.487 | 1.72 |  | 220 | 123.978 | 1.77 |
|  | 2D | 306 | 85.027 | 3.59 |  | 200 | 85.027 | 2.35 |
|  | 3D | 147 | 126.448 | 1.16 |  | 134 | 126.448 | 1.05 |
|  | 4D | 71 | 90.119 | 0.79 |  | 45 | 90.119 | 0.49 |
|  | 5D | 140 | 170.702 | 0.82 |  | 102 | 170.702 | 0.59 |
|  | 6D | 150 | 121.074 | 1.24 |  | 164 | 121.074 | 1.35 |
|  | 7D | 219 | 157.445 | 1.39 |  | 194 | 157.445 | 1.23 |
|  | Total | 1268 | 887.302 | 1.43 |  | 1059 | 874.793 | 1.21 |
| Whole genome | | 7714 | 2590.353 | 2.98 |  | 5873 | 2576.717 | 2.28 |

^a^Chromosome

Table S5. Linkage disequilibrium (LD) estimates in the subpopulation-I (SBP-I) and subpopulation-II (SBP-II).

| Genome | Chr^a^ |  | SBP-I |  |  |  | SBP-II |  |
| --- | --- | --- | --- | --- | --- | --- | --- | --- |
|  |  | Pairwise markers  ($r^{2}>$ 0.02) | $r^{2}$ mean ($r^{2}>$ 0.02) | LD ($r^{2}>$ 0.02) |  | Pairwise markers  ($r^{2}>$ 0.02) | $r^{2}$ mean ($r^{2}>$ 0.02) | LD ($r^{2}>$ 0.02) |
| A genome | 1A | 44902 (6.25%) | 0.10 | 5.15 |  | 15503 (4.40%) | 0.08 | 3.98 |
|  | 2A | 44089 (6.14%) | 0.10 | 3.98 |  | 28196 (8.01%) | 0.12 | 3.44 |
|  | 3A | 35231 (4.91%) | 0.09 | 9.21 |  | 12974 (3.68%) | 0.11 | 7.39 |
|  | 4A | 23995 (3.34%) | 0.11 | 8.15 |  | 10431 (2.96%) | 0.15 | 4.01 |
|  | 5A | 23686 (3.30%) | 0.09 | 3.58 |  | 9460 (2.69%) | 0.11 | 3.50 |
|  | 6A | 23656 (3.29%) | 0.10 | 4.55 |  | 15860 (4.50%) | 0.13 | 7.96 |
|  | 7A | 54748 (7.62%) | 0.09 | 5.11 |  | 38142 (10.83%) | 0.10 | 4.55 |
|  | Total | 250307 (34.85%) | 0.10 | 5.21 |  | 130566 (37.07%) | 0.11 | 4.77 |
| B genome | 1B | 51039 (7.11%) | 0.09 | 3.98 |  | 24501 (6.96%) | 0.09 | 4.55 |
|  | 2B | 89156 (12.41%) | 0.08 | 3.67 |  | 46066 (13.08%) | 0.10 | 4.56 |
|  | 3B | 82525 (11.49%) | 0.09 | 5.68 |  | 36685 (10.42%) | 0.09 | 5.15 |
|  | 4B | 9125 (1.27%) | 0.12 | 5.68 |  | 3837 (1.09%) | 0.10 | 5.09 |
|  | 5B | 67728 (9.43%) | 0.09 | 6.29 |  | 24784 (7.04%) | 0.08 | 5.11 |
|  | 6B | 59367 (8.27%) | 0.10 | 2.84 |  | 25267 (7.17%) | 0.09 | 2.27 |
|  | 7B | 50399 (7.02%) | 0.09 | 2.84 |  | 30301 (8.60%) | 0.09 | 2.85 |
|  | Total | 409339 (57.00%) | 0.09 | 3.93 |  | 191441 (54.36%) | 0.09 | 3.98 |
| D genome | 1D | 11962 (1.67%) | 0.16 | 7.96 |  | 7136 (2.03%) | 0.18 | 8.53 |
|  | 2D | 21518 (3.00%) | 0.24 | 9.55 |  | 6361 (1.81%) | 0.13 | 4.55 |
|  | 3D | 4012 (0.56%) | 0.16 | 6.83 |  | 3416 (0.97%) | 0.17 | 5.68 |
|  | 4D | 1136 (0.16%) | 0.17 | 3.98 |  | 577 (0.16%) | 0.35 | 1.14 |
|  | 5D | 3760 (0.52%) | 0.12 | 4.55 |  | 1556 (0.44%) | 0.14 | 3.41 |
|  | 6D | 4959 (0.69%) | 0.13 | 4.81 |  | 4703 (1.34%) | 0.14 | 5.95 |
|  | 7D | 11179 (1.56%) | 0.13 | 12.51 |  | 6429 (1.83%) | 0.18 | 13.22 |
|  | Total | 58526 (8.15%) | 0.18 | 10.81 |  | 30178 (8.57%) | 0.16 | 6.82 |
| Whole genome | | 718172 | 0.10 | 5.18 |  | 352185 | 0.11 | 4.62 |

^a^Chromosome; $r^{2}$is the squared correlation coefficient of the pairwise markers.
