# Supplementary material for: A classic approach for determining genomic prediction accuracy under terminal drought stress and well-watered conditions in wheat landraces and cultivars
Source: PLoS One. 2021 Mar 5;16(3):e0247824. doi: 10.1371/journal.pone.0247824 (PMC7935232; doi:10.1371/journal.pone.0247824)
Supplement: S5 File — (DOCX) [file pone.0247824.s005.docx]

Table S6. Descriptive statistics and variance parameters estimated for 16 agronomic traits studied in the association panel including 286 Iran breed wheat accessions grown under terminal drought stress (TDS) and well-watered (WW) conditions in semi-arid environments, Iran.

| Irrigation  conditions | Trait | Descriptive statistics | | |  | Variance parameters | | | |
| --- | --- | --- | --- | --- | --- | --- | --- | --- | --- |
|  |  | Min | Mean | Max |  | $\sigma_{G}^{2}$ | $\sigma_{G\times E}^{2}$ | $\sigma_{E}^{2}$ | $H^{2}$ |
| TDS | DTH | 158.00 | 199.68 | 230.00 |  | 43.22 | 11.72 | 3.57 | 0.86 |
|  | DTM | 192.00 | 246.33 | 276.00 |  | 11.51 | 24.65 | 2.08 | 0.47 |
|  | DHTM | 5.00 | 46.64 | 79.00 |  | 2.98 | 4.26 | 1.17 | 0.55 |
|  | PH | 49.00 | 104.45 | 151.00 |  | 17.99 | 11.19 | 0.76 | 0.76 |
|  | GY | 0.03 | 0.22 | 0.59 |  | 0.17 | 0.12 | 0.03 | 0.72 |
|  | TKW | 10.21 | 31.04 | 54.68 |  | 1.85 | 1.53 | 0.05 | 0.70 |
|  | SEL | 3.30 | 6.50 | 9.50 |  | 0.44 | - | 0.03 | - |
|  | SEW | 1.40 | 2.79 | 4.30 |  | 0.06 | - | 0.02 | - |
|  | SN | 12.00 | 41.15 | 86.00 |  | 2.17 | 2.08 | 1.12 | 0.62 |
|  | SPL | 4.20 | 10.11 | 15.70 |  | 2.02 | - | 0.12 | - |
|  | SPW | 0.20 | 2.13 | 6.10 |  | 0.51 | - | 0.03 | - |
|  | FLL | 9.50 | 22.41 | 37.00 |  | 10.72 | - | 0.55 | - |
|  | FLW | 3.50 | 9.44 | 20.35 |  | 5.39 | - | 0.41 | - |
|  | PL | 6.10 | 44.31 | 71.10 |  | 12.77 | - | 0.80 | - |
|  | SHD | 1.80 | 3.36 | 5.20 |  | 0.28 | - | 0.02 | - |
|  | AWL | 0.50 | 7.31 | 17.70 |  | 6.50 | - | 0.24 | - |
| WW | DTH | 156.00 | 199.94 | 230.00 |  | 43.30 | 11.89 | 3.12 | 0.87 |
|  | DTM | 210.00 | 252.13 | 280.00 |  | 16.84 | 29.68 | 1.76 | 0.52 |
|  | DHTM | 9.00 | 52.19 | 116.00 |  | 3.78 | 4.44 | 1.58 | 0.59 |
|  | PH | 64.00 | 109.66 | 164.00 |  | 25.57 | 12.41 | 1.11 | 0.80 |
|  | GY | 0.05 | 0.29 | 0.71 |  | 0.21 | 0.09 | 0.06 | 0.78 |
|  | TKW | 12.07 | 37.74 | 60.65 |  | 2.65 | 1.58 | 0.03 | 0.77 |
|  | SEL | 3.40 | 6.69 | 9.70 |  | 0.48 | - | 0.03 | - |
|  | SEW | 1.60 | 2.99 | 4.40 |  | 0.07 | - | 0.02 | - |
|  | SN | 13.00 | 43.67 | 88.00 |  | 3.68 | 2.79 | 1.58 | 0.67 |
|  | SPL | 5.00 | 10.60 | 15.90 |  | 2.59 | - | 0.12 | - |
|  | SPW | 0.70 | 3.32 | 6.40 |  | 0.55 | - | 0.04 | - |
|  | FLL | 13.00 | 26.37 | 41.60 |  | 11.06 | - | 0.68 | - |
|  | FLW | 4.50 | 10.18 | 20.50 |  | 5.51 | - | 0.45 | - |
|  | PL | 6.80 | 49.32 | 77.50 |  | 14.12 | - | 0.85 | - |
|  | SHD | 1.80 | 3.51 | 5.30 |  | 0.29 | - | 0.02 | - |
|  | AWL | 0.60 | 7.52 | 18.00 |  | 6.86 | - | 0.21 | - |

$\sigma_{G}^{2}$, genotype variance; $\sigma_{G\times E}^{2}$, genotype $\times$ environment variance; $\sigma_{E}^{2}$, residual variance;$H^{2}$, Heritability.

Table S7. Analysis of variance (ANOVA) for 16 agronomic traits in the association panel including 286 Iran bread wheat accessions grown under terminal drought stress (TDS) conditions in semi-arid environments, Iran.

| S.O.V | DF | Mean Square | | | | | | | | | | | | | | | |
| --- | --- | --- | --- | --- | --- | --- | --- | --- | --- | --- | --- | --- | --- | --- | --- | --- | --- |
|  |  | DTH | DTM | DHTM | PH | GY | TKW | SEL | SEW | SN | SPL | SPW | FLL | FLW | PL | SHD | AWL |
| Location | 1 | 351716.33** | 372313.85** | 293.04** | 110572.56** | 454.09** | 38601.74** | 1304.04** | 285.80** | 20327.65** | 343.75** | 208.34** | 7941.78** | 1815.11** | 15878.93** | 44.61** | 9.49** |
| Replication(Location) | 2 | 3.01 | 0.79 | 0.91 | 0.99 | 0.00 | 0.33 | 0.07 | 0.03 | 0.82 | 0.01 | 0.03 | 0.51 | 0.27 | 0.32 | 0.01 | 0.12 |
| Block(Replication/Location) | 84 | 3.44 | 2.19 | 5.35 | 0.74 | 0.03 | 0.50 | 0.03 | 0.02 | 5.43 | 0.18 | 0.04 | 0.63 | 0.40 | 0.72 | 0.03 | 0.31 |
| Genotype | 285 | 186.83** | 101.48** | 139.43** | 805.49** | 1.94** | 129.11** | 1.83** | 0.29** | 376.17** | 9.55** | 2.07** | 40.30** | 20.70** | 54.08** | 1.07** | 25.61** |
| Genotype*Location | 285 | 25.22** | 57.24** | 62.86** | 156.15** | 0.59** | 90.33** | 0.03^ns^ | 0.01^ns^ | 79.6** | 0.06^ns^ | 0.03^ns^ | 0.46^ns^ | 0.45^ns^ | 0.63^ns^ | 0.01^ns^ | 0.18^ns^ |
| Error | 486 | 3.42 | 2.06 | 5.39 | 0.77 | 0.03 | 0.50 | 0.03 | 0.02 | 1.05 | 0.14 | 0.04 | 0.60 | 0.41 | 0.81 | 0.03 | 0.27 |

DTH, days to heading; DTM, days to maturity; DHTM, duration of heading-to-maturity; PH, plant height (cm); GY, grain yield (kg/m^2^); TKW, thousand kernel weight (g); SEL, seed length (mm); SEW, seed width (mm); SN, seed number per spike (number); SPL, spike length (cm); SPW, spike weight (g); FLL, flag leaf length (cm); FLW, flag leaf width (mm); PL, peduncle length (cm); SHD, shoot diameter (mm) and AWL, awn length (cm). ^ns^ non-significant, ^*^ P $<$ 0.05 and ^**^ P $<$ 0.01.

Table S8. Analysis of variance (ANOVA) for 16 agronomic traits in the association panel including 286 Iran bread wheat accessions grown under well-watered (WW) conditions in semi-arid environments, Iran.

| S.O.V | DF | Mean Square | | | | | | | | | | | | | | | |
| --- | --- | --- | --- | --- | --- | --- | --- | --- | --- | --- | --- | --- | --- | --- | --- | --- | --- |
|  |  | DTH | DTM | DHTM | PH | GY | TKW | SEL | SEW | SN | SPL | SPW | FLL | FLW | PL | SHD | AWL |
| Location | 1 | 343073.18** | 405720.22** | 2625.25** | 86610.24** | 787.84** | 63980.99** | 1453.96** | 231.39** | 28565.10** | 69.12** | 1078.80** | 10528.30** | 1137.01** | 18434.63** | 27.63** | 43.78** |
| Replication(Location) | 2 | 1.66 | 3.43 | 7.75 | 2.04 | 0.11 | 0.01 | 0.02 | 0.02 | 1.01 | 0.03 | 0.08 | 0.11 | 0.81 | 0.21 | 0.05 | 0.20 |
| Block(Replication/Location) | 84 | 3.60 | 1.47 | 5.61 | 1.11 | 0.10 | 0.03 | 0.04 | 0.03 | 5.89 | 0.12 | 0.06 | 0.85 | 0.39 | 0.49 | 0.03 | 0.28 |
| Genotype | 285 | 189.29** | 98.87** | 153.02** | 769.83** | 2.62** | 130.05** | 1.68** | 0.28** | 445.76** | 9.72** | 1.91** | 41.14** | 20.22** | 83.76** | 1.07** | 24.15** |
| Genotype*Location | 285 | 25.12** | 46.41** | 59.62** | 142.38** | 0.61** | 92.31** | 0.02^ns^ | 0.01^ns^ | 65.80** | 0.10^ns^ | 0.03^ns^ | 0.49^ns^ | 0.24^ns^ | 0.65^ns^ | 0.01^ns^ | 0.13^ns^ |
| Error | 486 | 3.10 | 1.80 | 4.88 | 1.11 | 0.07 | 0.03 | 0.03 | 0.03 | 1.61 | 0.12 | 0.05 | 0.79 | 0.59 | 0.92 | 0.03 | 0.26 |

DTH, days to heading; DTM, days to maturity; DHTM, duration of heading-to-maturity; PH, plant height (cm); GY, grain yield (kg/m^2^); TKW, thousand kernel weight (g); SEL, seed length (mm); SEW, seed width (mm); SN, seed number per spike (number); SPL, spike length (cm); SPW, spike weight (g); FLL, flag leaf length (cm); FLW, flag leaf width (mm); PL, peduncle length (cm); SHD, shoot diameter (mm) and AWL, awn length (cm). ^ns^ non-significant, ^*^ P $<$ 0.05 and ^**^ P $<$ 0.01.

Table S9. Pearson correlation coefficients for 16 agronomic traits in the association panel including 286 Iran bread wheat accessions under terminal drought stress (TDS) conditions in semi-arid environments, Iran.

| Trait | DTH | DTM | DHTM | PH | GY | TKW | SEL | SEW | SN | SPL | SPW | FLL | FLW | PL | SHD | AWL |
| --- | --- | --- | --- | --- | --- | --- | --- | --- | --- | --- | --- | --- | --- | --- | --- | --- |
| DTH | 1 |  |  |  |  |  |  |  |  |  |  |  |  |  |  |  |
| DTM | 0.29** | 1 |  |  |  |  |  |  |  |  |  |  |  |  |  |  |
| DHTM | -0.66** | 0.43** | 1 |  |  |  |  |  |  |  |  |  |  |  |  |  |
| PH | 0.61** | 0.12* | -0.40** | 1 |  |  |  |  |  |  |  |  |  |  |  |  |
| GY | 0.12* | 0.11* | 0.04 | -0.06 | 1 |  |  |  |  |  |  |  |  |  |  |  |
| TKW | -0.14* | 0.10* | 0.07 | -0.08 | 0.18** | 1 |  |  |  |  |  |  |  |  |  |  |
| SEL | -0.17** | -0.19** | 0.12* | 0.15* | 0.12* | 0.14* | 1 |  |  |  |  |  |  |  |  |  |
| SEW | 0.18** | 0.15* | -0.03 | 0.09 | 0.14* | 0.10* | 0.17** | 1 |  |  |  |  |  |  |  |  |
| SN | 0.07 | -0.08 | 0.04 | -0.00 | 0.17** | 0.05 | -0.06 | 0.08 | 1 |  |  |  |  |  |  |  |
| SPL | -0.09 | -0.12* | -0.07 | -0.09 | -0.08 | -0.04 | 0.09 | -0.06 | 0.09 | 1 |  |  |  |  |  |  |
| SPW | 0.10* | 0.11* | -0.03 | -0.05 | 0.16* | 0.11* | -0.19** | 0.12* | 0.17** | 0.10* | 1 |  |  |  |  |  |
| FLL | -0.13* | -0.14* | 0.10* | -0.08 | 0.12* | -0.07 | 0.11* | 0.15* | -0.10* | -0.09 | 0.12* | 1 |  |  |  |  |
| FLW | -0.09 | -0.10* | -0.02 | -0.03 | -0.11* | -0.09 | -0.10* | 0.11* | 0.12* | -0.07 | 0.14* | 0.11* | 1 |  |  |  |
| PL | -0.08 | 0.08 | -0.09 | 0.10* | -0.07 | 0.05 | -0.09 | -0.10* | -0.06 | -0.06 | -0.07 | -0.08 | -0.09 | 1 |  |  |
| SHD | 0.07 | -0.09 | 0.11* | -0.02 | 0.10* | -0.06 | 0.04 | -0.10* | 0.11* | 0.07 | 0.12* | 0.10* | 0.16* | -0.03 | 1 |  |
| AWL | -0.00 | 0.00 | 0.01 | -0.03 | 0.01 | 0.02 | 0.00 | 0.00 | 0.02 | -0.00 | 0.04 | 0.06 | 0.03 | 0.01 | -0.01 | 1 |

DTH, days to heading; DTM, days to maturity; DHTM, duration of heading-to-maturity; PH, plant height (cm); GY, grain yield (kg/m^2^); TKW, thousand kernel weight (g); SEL, seed length (mm); SEW, seed width (mm); SN, seed number per spike (number); SPL, spike length (cm); SPW, spike weight (g); FLL, flag leaf length (cm); FLW, flag leaf width (mm); PL, peduncle length (cm); SHD, shoot diameter (mm) and AWL, awn length (cm). ^*^ P $<$ 0.05 and ^**^ P $<$ 0.01.

Table S10. Pearson correlation coefficients for 16 agronomic traits in the association panel including 286 Iran bread wheat accessions under well-watered (WW) conditions in semi-arid environments, Iran.

| Trait | DTH | DTM | DHTM | PH | GY | TKW | SEL | SEW | SN | SPL | SPW | FLL | FLW | PL | SHD | AWL |
| --- | --- | --- | --- | --- | --- | --- | --- | --- | --- | --- | --- | --- | --- | --- | --- | --- |
| DTH | 1 |  |  |  |  |  |  |  |  |  |  |  |  |  |  |  |
| DTM | 0.37** | 1 |  |  |  |  |  |  |  |  |  |  |  |  |  |  |
| DHTM | -0.71** | 0.48** | 1 |  |  |  |  |  |  |  |  |  |  |  |  |  |
| PH | 0.65** | 0.26** | -0.45** | 1 |  |  |  |  |  |  |  |  |  |  |  |  |
| GY | 0.21** | -0.27** | 0.19** | 0.09 | 1 |  |  |  |  |  |  |  |  |  |  |  |
| TKW | 0.32** | 0.30** | -0.18** | -0.14* | 0.30** | 1 |  |  |  |  |  |  |  |  |  |  |
| SEL | -0.28** | 0.35** | -0.07 | -0.21** | 0.24** | 0.35** | 1 |  |  |  |  |  |  |  |  |  |
| SEW | 0.30** | -0.37** | 0.08 | 0.12* | -0.26** | 0.25** | 0.36** | 1 |  |  |  |  |  |  |  |  |
| SN | -0.12* | -0.14* | 0.17** | -0.01 | 0.33** | -0.09 | 0.12* | 0.12* | 1 |  |  |  |  |  |  |  |
| SPL | 0.09 | -0.08 | -0.05 | 0.11* | -0.10* | -0.03 | -0.11* | -0.04 | 0.12* | 1 |  |  |  |  |  |  |
| SPW | 0.32** | 0.28** | 0.08 | 0.16* | 0.27** | 0.26** | -0.31** | 0.34** | 0.30** | 0.10* | 1 |  |  |  |  |  |
| FLL | -0.21** | -0.26** | -0.24** | 0.18** | 0.26** | -0.19** | 0.27** | -0.24** | 0.16* | 0.12* | 0.25** | 1 |  |  |  |  |
| FLW | -0.14* | -0.17** | -0.05 | -0.05 | -0.20** | 0.12* | -0.12* | -0.14* | 0.19** | -0.09 | -0.20** | 0.20** | 1 |  |  |  |
| PL | -0.15* | 0.13* | -0.10* | -0.11* | -0.13* | -0.12* | 0.17** | -0.13* | -0.13* | -0.08 | 0.16* | 0.18** | 0.15* | 1 |  |  |
| SHD | -0.10* | -0.10* | 0.12* | 0.04 | 0.16* | -0.09 | 0.08 | -0.11* | 0.17** | 0.10* | 0.17** | -0.15* | 0.18** | -0.05 | 1 |  |
| AWL | 0.01 | 0.02 | 0.07* | -0.15* | 0.10* | 0.11* | 0.04 | 0.01 | -0.05 | -0.01 | 0.08 | 0.16* | 0.06 | 0.12* | -0.02 | 1 |

DTH, days to heading; DTM, days to maturity; DHTM, duration of heading-to-maturity; PH, plant height (cm); GY, grain yield (kg/m^2^); TKW, thousand kernel weight (g); SEL, seed length (mm); SEW, seed width (mm); SN, seed number per spike (number); SPL, spike length (cm); SPW, spike weight (g); FLL, flag leaf length (cm); FLW, flag leaf width (mm); PL, peduncle length (cm); SHD, shoot diameter (mm) and AWL, awn length (cm). ^*^ P $<$ 0.05 and ^**^ P $<$ 0.01.
