# Supplementary material for: A classic approach for determining genomic prediction accuracy under terminal drought stress and well-watered conditions in wheat landraces and cultivars
Source: PLoS One. 2021 Mar 5;16(3):e0247824. doi: 10.1371/journal.pone.0247824 (PMC7935232; doi:10.1371/journal.pone.0247824)
Supplement: S6 File — (DOCX) [file pone.0247824.s006.docx]

Table S11. Comparison of three genome-wide association study (GWAS) models used to discover significant markers under terminal drought stress (TDS) and well-watered (WW) conditions in the association panel including 286 Iran bread wheat accessions.

| Model | Used threshold  ($P$ value $\leq$) | TDS |  | WW |
| --- | --- | --- | --- | --- |
|  |  | Number of  identified markers |  | Number of  identified markers |
| K | 0.001 | 79 |  | 93 |
|  | 0.01 | 1047 |  | 1086 |
|  | 0.05 | 6332 |  | 6425 |
| K$+$Q | 0.001 | 82 |  | 105 |
|  | 0.01 | 1091 |  | 1109 |
|  | 0.05 | 6546 |  | 6576 |
| K$+$PCA | 0.001 | 89 |  | 95 |
|  | 0.01 | 1110 |  | 1130 |
|  | 0.05 | 6550 |  | 6484 |

The GWAS models were based on the mixed linear model (MLM). The estimates belong to sixteen agronomic traits.
