# Supplementary material for: A classic approach for determining genomic prediction accuracy under terminal drought stress and well-watered conditions in wheat landraces and cultivars
Source: PLoS One. 2021 Mar 5;16(3):e0247824. doi: 10.1371/journal.pone.0247824 (PMC7935232; doi:10.1371/journal.pone.0247824)
Supplement: S8 File — (DOCX) [file pone.0247824.s008.docx]

Table S14. A description of markers selected for 16 agronomic traits in the association panel including 286 Iran bread wheat accessions grown under terminal drought stress (TDS) and well-watered (WW) conditions in semi-arid environments, Iran.

| Trait | Marker^a^ | Marker sequence | Marker start  position (bp) | Predicted  candidate gene | Candidate gene  start position (bp) | Molecular function and  biological process |
| --- | --- | --- | --- | --- | --- | --- |
| DTH | rs48214 | TGCAGGCGCTGGCTCAGCCACGCCTCCGATCG CGCTTTCCTCCGCCGTTTCCGCGAGCTCCACC | 740,540,882 | TraesCS3B02G497100 | 740,540,681 | Protein binding, F-box-like domain superfamily |
| DTM | rs34236 | TGCAGCTAAAACATGAAGGAAACCCACGAAGA TCCACACCAGAGCATGCAAGATCATAAAACTC | 617,875,979 | TraesCS2B02G430000 | 617,929,704 | Transmembrane transporter activity, Sugar phosphate transporter |
|  | rs28367 | TGCAGCGACGCAACCACCGAAATAATGCGCAC AAGTACGACGCAGCAACCCTACCGAAATAATA | 29,263,662 | TraesCS2A02G065000 | 29,285,160 | Prostaglandin-E synthase activity, Glutaredoxin active site |
|  | rs61739 | TGCAGTGGTAGTATATCTTCTTCGCAAGATGGT TCACGTGTTACGAGTAGTATAGAGGATACGG | 37,575,079 | TraesCS4B02G049400 | 37,848,176 | Embryo development ending in seed dormancy, Maintenance of protein localization in endoplasmic reticulum |
| DHTM | rs41211 | TGCAGCTTGACACGCTGGTGATGGTCGTCTTC TTCTTCTCGCTCCATCCAGAAATTGTTTATCG | 717,127,183 | TraesCS2B02G521800 | 716,809,651 | Protein kinase domain, Protein phosphorylation, Serine/threonine protein kinase, Hyperosmotic stress respons |
|  | rs19295 | TGCAGCATCCTCTGCACTCCGCGAGAACACAAA TCCTAACAGGCAGCCATCGAGAAAGGAAACA | 570,791,395 | TraesCS5A02G372600 | 570,791,269 | Integral component of membrane, Zinc finger, RING/FYVE/PHD-type |
|  | rs31147 | TGCAGCGCTGAATAGGCGGAGCTCCTGTTCGC CGCCCGCGAGCGGCAAACGATTGAGCCTTCGC | 625,068,771 | TraesCS7A02G430600 | 624,964,931 | Heat shock protein 70 family |
| PH | rs1184 | TGCAGAACAGCTGTAGACGAGATGGATGCTAG TCTCGCTATAAGCGAGACATAGCATTCACGGT | 101,069,372 | TraesCS2B02G134200 | 100,334,620 | Putative indole-3-acetic acid-amido synthetase, GH3 family |
| GY | rs65502 | TGCAGTTTTTCAGTGAGGAGGAGCTGATGTGG CTCCCAAGATTTCTCAATGACAGATTACACAG | 595,132,862 | TraesCS5B02G419500 | 595,121,952 | Serine-type endopeptidase inhibitor activity, Response to wounding, Proteinase inhibitor |
| TKW | rs54576 | TGCAGGTGTCGAACAACCAATAGAATGTAGAA GGGAGTTGCTGGCATAATCCAGAATTTGATGC | 70,871,631 | TraesCS2A02G120900 | 70,871,585 | Protein binding, Tubby, C-terminal, F-box-like domain superfamily |
|  | rs17145 | TGCAGCAGCGCCTTAAGCTGGTCCTGCGCCAGG GCCTTGGTCGGGAACACGTAGAGCGCGCAGG | 15,865,937 | TraesCS6A02G031400 | 15,863,959 | ATP binding, DEAD/DEAH-box helicase, P-loop containing nucleoside triphosphate hydrolase |
| SEL | rs998 | TGCAGAAATTCGACCCCGAACCCAACCAAATC TACCTACGCGACCCGCAGAGCAGAACCCCAAA | 498954562 | TraesCS6A02G272000 | 498,950,805 | - |
| SEW | rs55852 | TGCAGTAAGCTTGAGTCGGTCGGCCGAGTAGG TCACACACACGGCGCTCGCCAAATTAGCCCAC | 758,346,322 | TraesCS3B02G513400 | 756,628,146 | Methyltransferase activity, SAM dependent carboxyl methyltransferase |
| SN | rs51365 | TGCAGGGGTCCATGCCGCTGCACAGCCGTCGT CTCTCTTGGGTCAGCCACTTCCGCATGTCGCC | 20,691,420 | TraesCS2D02G053200 | 20,676,303 | Oxidoreductase activity, Arsenite methyltransferase-like, SAM dependent methyltransferase, FAD/NAD(P)-binding domain superfamily |
|  | rs65502 | TGCAGTTTTTCAGTGAGGAGGAGCTGATGTGG CTCCCAAGATTTCTCAATGACAGATTACACAG | 595,132,862 | TraesCS5B02G419500 | 595,121,952 | Serine-type endopeptidase inhibitor activity, Response to wounding, Proteinase inhibitor |
|  | rs64054 | TGCAGTTGCCAACACCAACCTCACTGTCCTCG TCAAGCGCCCCCATCGTCGTTCAACAGTACCG | 94,172,323 | TraesCS7A02G142600 | 94,186,617 | DNA binding, Protein dimerization activity, Transcription factor, MADS-box superfamily |
| SPL | rs60932 | TGCAGTGCGCTCTACTTGGCCATTTCGGGTCAC CGCCTCACCGAGATCGGAAGAGCGGGATCAC | 550,111,479 | TraesCS3A02G311500 | 550,632,747 | RNA-directed 5'-3' RNA polymerase activity |
| SPW | rs15276 | TGCAGCACGTCCCGCATGCAGCGGGAGTGAACA GGAACAACAGGCCGAGATCGGAAGAGCGGGA | 338,834,928 | TraesCS1A02G187400 | 338,581,948 | Catalytic activity, Phospholipase D/Transphosphatidylase, C2 domain superfamily |
|  | rs2368 | TGCAGAAGTGGAGCTAGTGCAGCACGTCCTAG GTGGGTCGGCCGACTTGTCGTGCTGCTGTCCG | 46,389,940 | TraesCS1B02G063500 | 48,267,261 | Poly(A)+ mRNA export from nucleus, GLE1-like superfamily |
|  | rs65502 | TGCAGTTTTTCAGTGAGGAGGAGCTGATGTGG CTCCCAAGATTTCTCAATGACAGATTACACAG | 595,132,862 | TraesCS5B02G419500 | 595,121,952 | Serine-type endopeptidase inhibitor activity, Response to wounding, Proteinase inhibitor |
|  | rs53982 | TGCAGGTGCCGTGCGCCGTCTCCGCGCGCCAC GCCGCAGGGACCACAAGTTTGAGCTTCGACAG | 504,507,751 | TraesCS5D02G460700 | 505,128,032 | Iron ion binding, Oxidoreductase activity, L-ascorbic acid binding, Prolyl 4-hydroxylase, alpha subunit |
| FLL | rs58293 | TGCAGTCCATCACGCGGTTCTGTCTGTCTTGGG ATCGACTCGACGCAGCACCGACCGTCTACAT | 132,328,614 | TraesCS4B02G116600 | 134,589,796 | TB2/DP1/HVA22-related protein |
|  | rs59732 | TGCAGTCTTGCAGCTAGCTCGTGGAACGAGCGG CCCACCGAGATCGGAAGAGCGGGATCACCGA | 660,491,056 | TraesCS6B02G384900 | 659,639,554 | Lipid transporter activity, RFT1 |
| FLW | rs6770 | TGCAGAGGCTGCATCCTCTCCAGCTTCGCCTCG GCCGCGGGGAGCGCCGCCGCCTCCTCCGAGA | 70,866,014 | TraesCS3B02G104400 | 70,865,752 | Nucleotide binding, Ligase activity, tRNA aminoacylation for protein translation, Aspartyl/Asparaginyl-tRNA synthetase, GAD-like domain superfamily, Aminoacyl-tRNA synthetase, Cass II (D/K/N), OB-fold nucleic acid binding domain |
| PL | rs31423 | TGCAGCGGAGCATAAGTGGCACCGCCTCCTTG GTCATATTAAAATCGGAGCATGTGATGGGCAG | 56,262,500 | TraesCS7A02G092300 | 56,151,832 | NADP binding, Phosphogluconate dehydrogenase (decarboxylating) activity, D-gluconate metabolic process, Pentose-phosphate shunt, 6-phosphogluconate dehydrogenase, domain 2, NAD(P)-binding domain superfamily |
| SHD | rs11489 | TGCAGCAACCCAGCGAGTGGTTGGTTTGTACC CCGAGGCCTCCACGTTGGGGGGCTTGGCCGCT | 719,976,174 | TraesCS4A02G456300 | 721,380,650 | Cellulose synthase (UDP-forming) activity, Cellulose biosynthetic process, Cell wall organization, Zinc finger, RING/FYVE/PHD-type, Nucleotide-diphospho-sugar transferases |
|  | rs49193 | TGCAGGCTGGGGCAACATGGGTGGCGGTGCCA CACAGCTCATCATGCCGCTTGTCTTCCACGCA | 15,766,486 | TraesCS6A02G031100 | 15,765,759 | Tansmembrane transport, Major facilitator superfamily |
| AWL | rs59275 | TGCAGTCGTGCGGATATTCGATCTGGCCGCAG GCGCAACTGGAAATCACGTTGCATCCCCGTGG | 15,766,486 | TraesCS6A02G032600 | 16,226,818 | Protein binding, RNA processing, Regulation of timing of transition from vegetative to reproductive phase, HAT (Half-A-TPR) repeat, Tetratricopeptide-like helical domain superfamily |
|  | rs8958 | TGCAGATGAATCTTGTGGTGGAGGCGCGGGTG ACGCTGGACGAGGCGTGGGCGGCGCTCGGGGA | 41,407,773 | TraesCS5B02G037500 | 41,827,752 | Microtubule motor activity, ATP binding, Kinesin motor domain superfamily, P-loop containing nucleoside triphosphate hydrolase |

^a^Marker ID. Marker start position (bp), predicted candidate gene, candidate gene start position (bp), molecular function, and biological process are obtained from http://plants.ensembl.org and https://triticeaetoolbox.org. DTH, days to heading; DTM, days to maturity; DHTM, duration of heading-to-maturity; PH, plant height; GY, grain yield; TKW, thousand kernel weight; SEL, seed length; SEW, seed width; SN, seed number per spike; SPL, spike length; SPW, spike weight; FLL, flag leaf length; FLW, flag leaf width; PL, peduncle length; SHD, shoot diameter and AWL, awn length.
